# A portable primary radar for general aviation

**DOI:** 10.1371/journal.pone.0239892

**Published:** 2020-10-01

**Authors:** Jerom Maas, Ronald van Gent, Jacco Hoekstra

**Affiliations:** 1 Control & Operations, Delft University of Technology, Delft, The Netherlands; 2 Selfly B.V, Soest, The Netherlands; Oak Ridge National Laboratory, UNITED STATES

## Abstract

A detailed situation awareness of the local environment is essential for safe flight in General Aviation. When operating under Visual Flight Rules, eyesight is crucial for maintaining situation awareness and objects may be overlooked. Technical solutions such as Flarm have been sought, but they only work on a basis of co-operation: obstacles without the proper equipment are invisible. Recent developments in the field of radar technology, partly empowered by the demand for sensors for autonomous cars, have improved the size and power consumption of available hardware. Today, the hardware exists to build a portable primary radar system for situation awareness. In this paper the results are presented of efforts to build the first portable primary radar for general, which has to be lightweight, cheap and have a low power consumption. The focus in this paper is on the software design of such a radar system. The physical principles of radar sensing are described, as well as the scientific steps needed to provide situation awareness. The hardware and software for the radar are both built and tested, and the results of these tests are presented. A flight experiment is performed with a small aircraft flying past a stationary radar on a small hill. It is found that the radar is capable of detecting the aircraft up to a distance of at least 3 kilometers. 3D localization is performed and the location determined by the radar was on average 46 meters away from the aircraft position as measured by satellite navigation, relative to a total distance of about 1000 meters from the radar. A low-pass filter can be applied on the raw results in order to improve the location estimation further. Future research will focus on bringing the portable radar in motion while operating.

## Introduction

In order to guarantee safe flight, it is essential to be aware of the environment around the aircraft in aviation. Lethal collisions can happen in General Aviation (GA) when pilots flying under Visual Flight Rules (VFR) are confronted with unexpected Instrument Meteorological Conditions (IMC), which limit the vision of a pilot [1, 2]. The detection of hazards around the aircraft may also be hindered by glare from the sun, the position of the own wings, or the size and attitude of the object [3]. On top of that, the development of Unmanned Aerial Systems (UAS) is expected to lead to an increase in traffic in uncontrolled airspace, where conflicts between partakers will occur more and more frequently [4]. Air Traffic Control (ATC) may not be present to guarantee safety, according to the plans for the future development of air traffic management [5, 6]. Therefore, reliable local methods for providing situation awareness are required.

Technical solutions have been sought to improve situation awareness. Devices such as Traffic Collision Avoidance System (TCAS) and Flarm can give pilots proximity warnings and even resolution advisories [7, 8], but the systems can be expensive, specifically for GA. Moreover: these systems are transponder-based and are therefore dependent on the presence of a transponder in the target aircraft. Towers, mountains and aircraft that do not carry the proper hardware are invisible for these systems and a pilot relying on them may be tricked by a false sense of safety. An independent solution for objects surveillance in an aircraft’s vicinity has not yet been found.

A hypothetical ideal solution would be to take a high-tech version of an airport surveillance radar on board of an aircraft. These systems, of which the first were built in the 1950’s, can independently detect a multitude of objects around an airport, whether transponder-equipped or not. Tuning of ground radar systems can empower them to observe even birds or rain clouds. With a system like this, the situational awareness of a pilot could be enhanced to a great extent. However, airport surveillance radars are too big and heavy to be carried on board of GA aircraft, and they consume more power than what a typical GA aircraft can provide. Also, the price of such a system is too steep to be considered for a regular GA aircraft owner. Moreover: these radars only provide 2D information about objects; altitude information is usually gathered by the aircraft transponder in Mode C or Mode S, for which a Secondary Surveillance Radar is required. Therefore, airport surveillance radars are unsuitable for taking on board of GA aircraft.

But recent developments in radar hardware have improved the specifications to a point where it may be possible build small low-power radar systems. In the 1970’s, marine radars have been introduced that can be taken on board of boats to improve the situational awareness, and in the early 2010’s, bird radar systems have been designed and built at airports. The present-day interest in self-driving cars have instigated a renewed focus on radar sensing [9–11]. This applies to hardware manufacturers that aim to improve specifications such as accuracy, weight and power consumption for a better cost, and it applies to scientists that use modern computational power to find new data processing algorithms to improve the results [12]. DIY-radio hardware that can match professional Automatic Dependent Surveillance Broadcast (ADS-B) receivers can be bought for use at home for less than the price of a computer, and the size of radar antennas is small enough to be fitted behind the front bumper of a self-driving car [9, 11]. It has become possible to design hardware for sense-and-avoid purposes on board of a GA aircraft.

This new hardware also brings new scientific challenges, since aviation is different from road traffic or shipping. A notable difference is the presence of a vertical dimension: while road traffic and shipping take place on the surface of the earth—an approximate two-dimensional plane—aviation is performed in the three-dimensional airspace above it. This means that objects of interest for the pilot can come from many different directions and that for any object, its location must therefore be determined in three dimensions. This task is complicated by the attitude of the aircraft itself, which can vary along three axes as well. Also, the presence of driving lanes cannot be assumed in aviation. This means that simplifications that are useful in head-tail collision prevention for car traffic, will not hold in flight. Further, aviation takes place on a larger scale than road traffic, with larger distances and higher velocities. This will not impact the theoretical limits since better hardware can be bought to overcome larger distances, but advanced software may be necessary to improve accuracy in order to keep the hardware affordable for GA aircraft. These examples show that new research is required before sense-and-avoid radar systems can be put into use in aviation.

In this paper, the results are presented of multiple experiments that work towards the goal of developing a portable radar system for local surveillance. The focus of this paper is on the detection of objects in the radar output, and on finding their locations in 3D space. In section 1, the hardware used in this research is described and the relevant theory is introduced. The algorithm for detecting the object pixels in the radar image is described in section 2, and the strategy of three-dimensional localization is described in section 3. These two chapters are illustrated on the basis of a simple static experiment, but a dynamic experiment is also carried out to assess the performance of the radar. This experiment is described in section 4, and its results are presented in section 5. A discussion about the results can be found in section 6, and conclusions about the experiments are found in section 7.

## 1 Hardware

In this section, the hardware used in this research is described, as well as the theoretic principles that form its scientific foundation. Three sub-sections are used for this. The theoretic principles and the resulting radar image are introduced in section 1.1. The issue of aliasing is introduced in section 1.2. In section 1.3, an overview is given for the steps necessary for object detection in GA.

**Fig 1:**
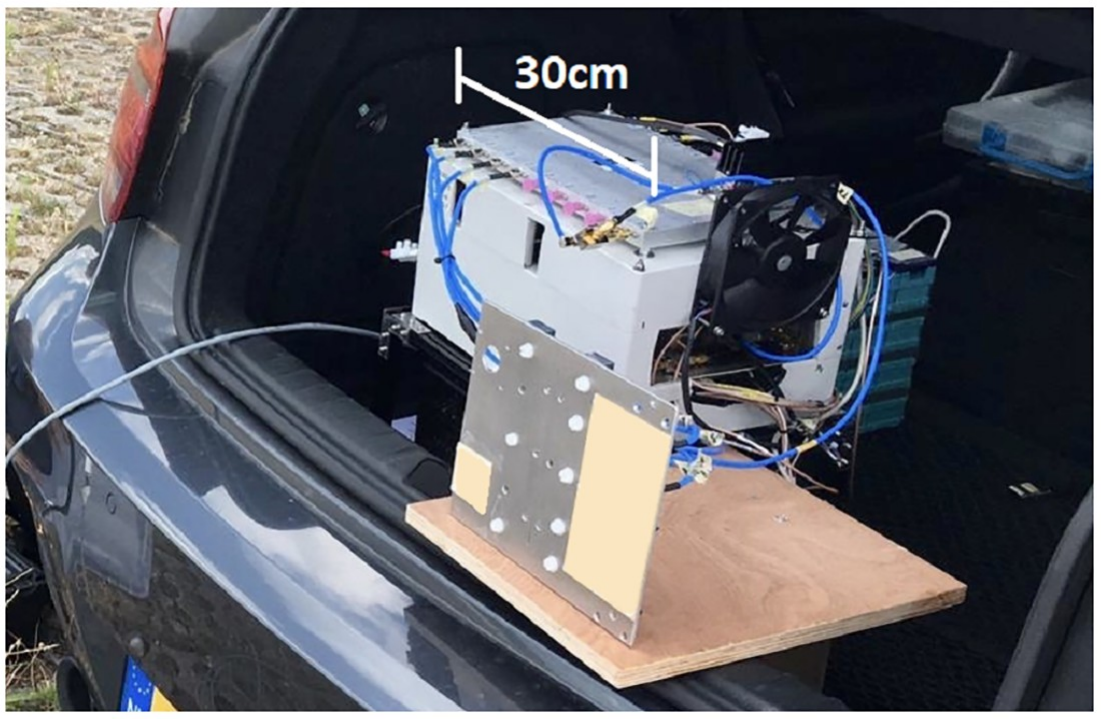
The hardware setup used in this research.

The radar is constructed by the company MetaSensing Radar Solutions in Noordwijk, the Netherlands. Its technical specifications are listed in Table 1. An image of the hardware used in this research is seen in Fig 1. The radar and its power supply fit within the trunk of a passenger car, as seen in the image. In this research, the radar was always operated from within the trunk of this car.

**Table 1:** Technical specifications of the radar hardware.

Parameter	Value
Carrier Frequency	9.425 *GHz*
Wavelength	31.83 *mm*
Sampling Frequency	10 *MHz*
Pulse Repetition Frequency	4921 *Hz*
Power Emitted	40 *dBm*
Bandwidth	10 *MHz*

### 1.1 Principles of FMCW radar

**Fig 2:**
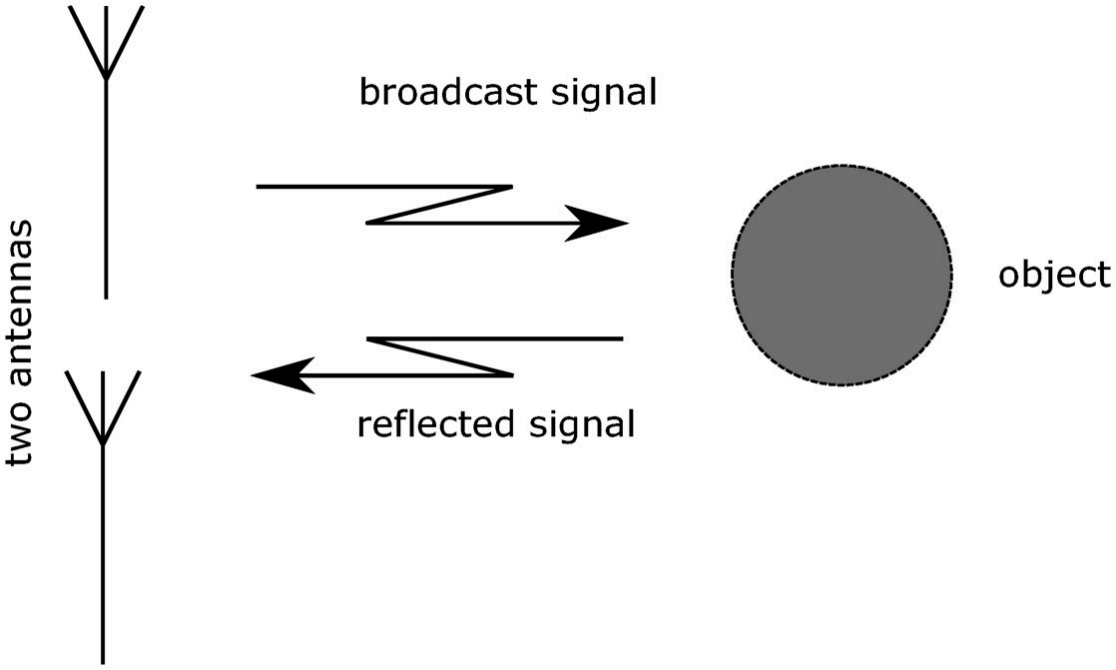
Transmission and reflection of a radar signal.

**Fig 3:**
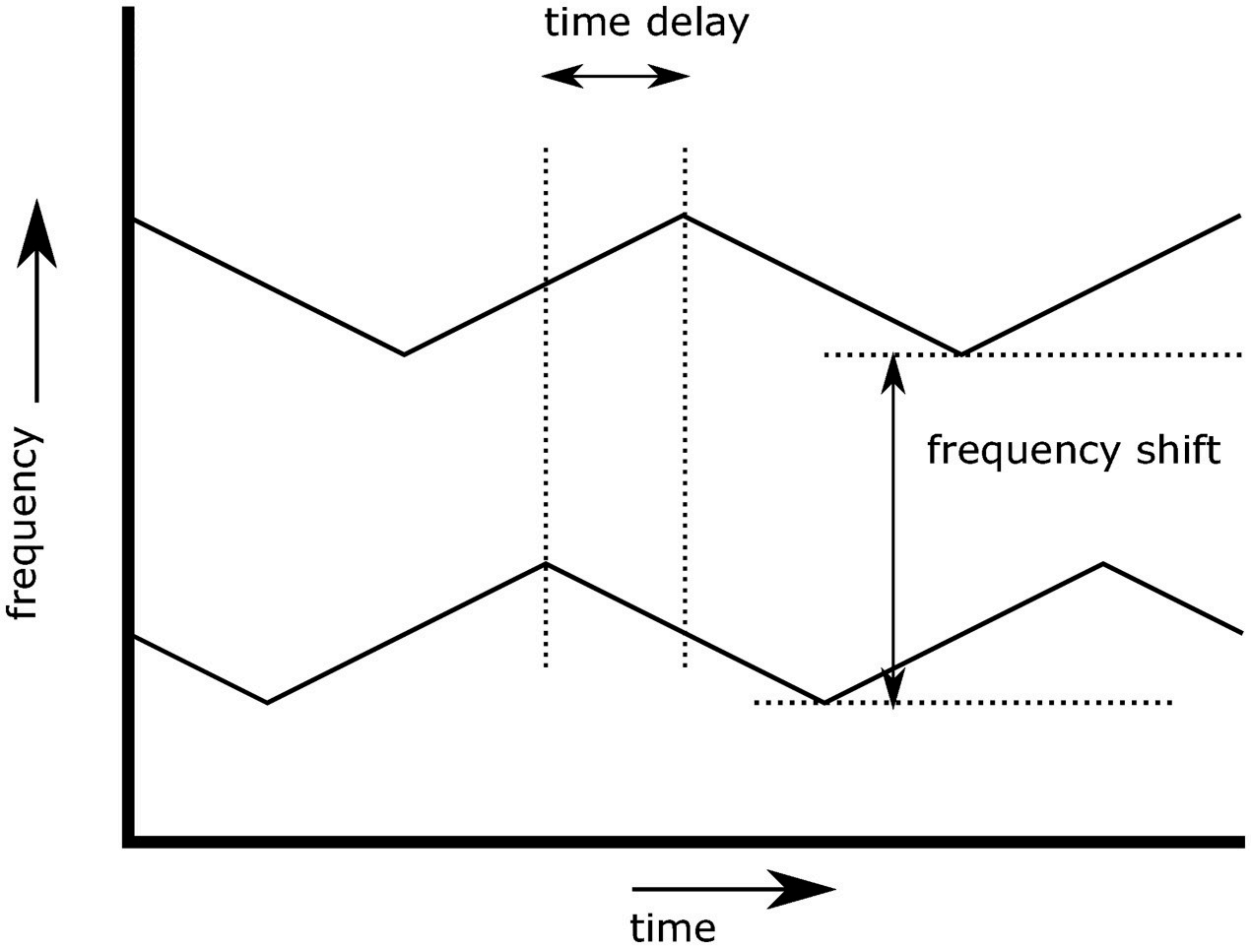
Differences between transmitted and received signals.

A modern Frequency Modulated Continuous Wave (FMCW) radar is used for this research. These radars transmit a non-stop signal, of which the frequency is varied around a central value. The signals are broadcast to the surroundings of the radar, reflect on present surfaces and are received again by the antennas of the system. The received signal is compared to the transmitted one, and the differences can be used to compute the time delay and Doppler shifts of the received signal. From these, the distance to the target and the radial velocity of the target can be found, respectively. [13, 14]. This principle is illustrated in Figs 2 and 3. FMCW radars can be built with inexpensive hardware, since the frequencies which are observed are lower. Because a continuous signal is transmitted, the power consumption of an FMCW radar is lower than that of a pulse radar. These properties make FMCW radar suitable for GA applications.

As can be seen in Fig 3, the shape of the frequency modulation facilitates a comparison between the broadcast and received signal. The time shift that is found can be used to compute the range to the object. Since the radar signals travel with the speed of light, the range to the object (*R*) can be found with Eq 1, in which Δ*t* is the time difference and *c* the speed of light.

$$\begin{array}{c} R=\frac{c\cdot \Delta t}{2} \end{array}$$ (1)

The vertical shift between the original and received signal is a consequence of the Doppler effect. This is caused by the objects moving relative to each other in longitudinal direction. Therefore, if the object is moving towards the radar or away from the radar, this will be visible in the Doppler results. A sideways movement will not result in a Doppler shift. Since the velocity of moving objects is negligible with respect to the radio propagation speed, the radial velocity (*V*_*R*_) can be found using Eq 2.

$$\begin{array}{c} V_{R}=\frac{\Delta f}{f_{0}}\cdot c \end{array}$$ (2)

In Eq 2, the frequency difference is denoted by Δ*f* and *f*_0_ is the transmitted center frequency.

**Fig 4:**
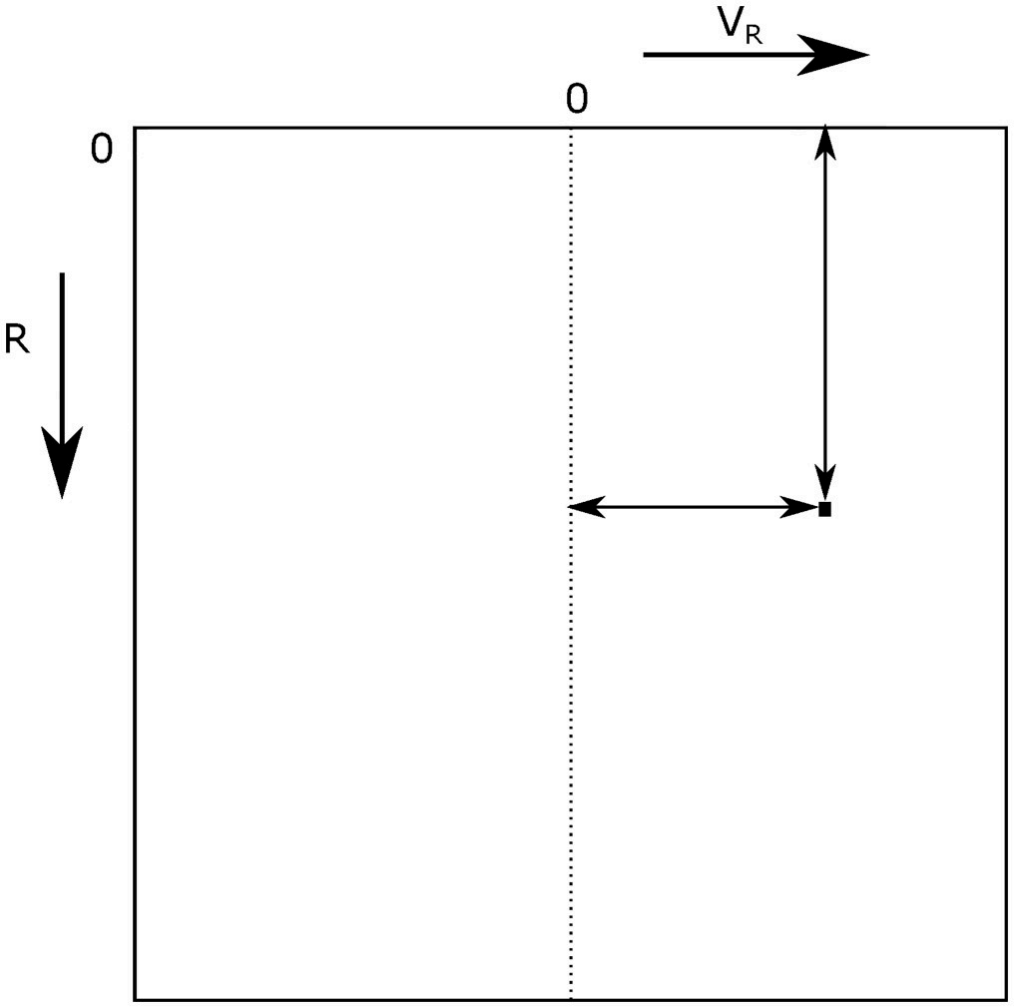
The axes of a radar image and how to read pixel coordinates.

When operating, the radar will receive a multitude of reflections from surfaces in its vicinity. All these reflections are sensed by the same antenna, so the resulting input signal is an addition of all reflections. The input signal is converted from analog to digital and a Fourier analysis is performed to reconstruct the reflections. For each of the components of the Fourier result, the values for *R* and *V*_*R*_ are found as well as the amplitude of the sinusoid. These three parameters are used to construct a greyscale radar image, in which *R* and *V*_*R*_ form the pixel coordinates, and the intensity of the signal is used for the pixel intensity. An illustration of a radar image can be found in Fig 4, in which the axes are illustrated, as well as the way how to find the *R* and *V*_*R*_ values of a pixel of interest.

### 1.2 Aliasing

**Fig 5:**
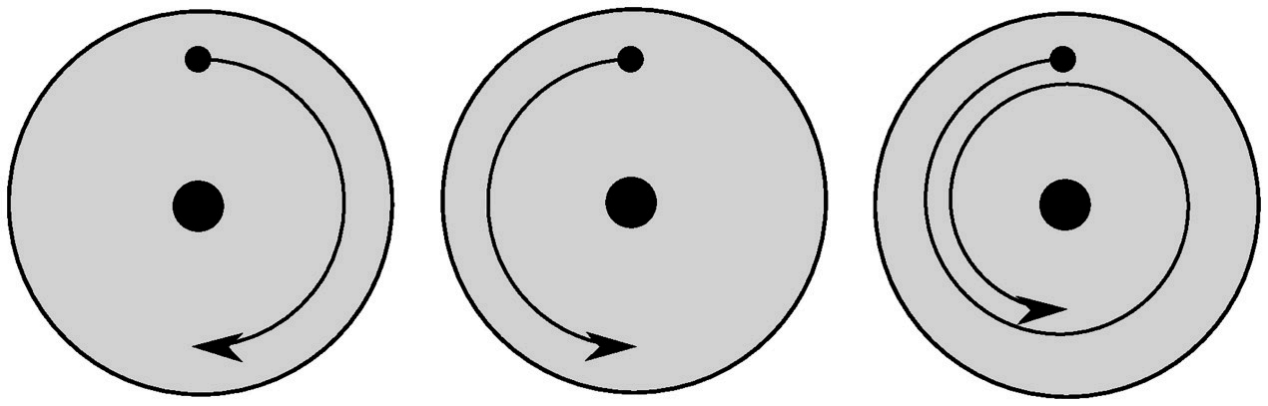
Three rotating discs that are observed to be equal when sampled at the correct frequency.

When an analog signal is sampled and converted to digital values, it is impossible to determine the exact original frequency. This is because of the phenomenon of aliasing. This means that two sinusoidal signals which differ in frequency with an exact amount, can not be separated from one another. This can be illustrated with an example of a moving disc, as can be seen in Fig 5. In this example, three discs rotate with different rotational velocities. If a picture is taken of these discs at the right moment, when they performed half a revolution clockwise or counterclockwise, the pictures will be identical, and it is not possible to determine the rotational velocity uniquely. [15]

**Fig 6:**
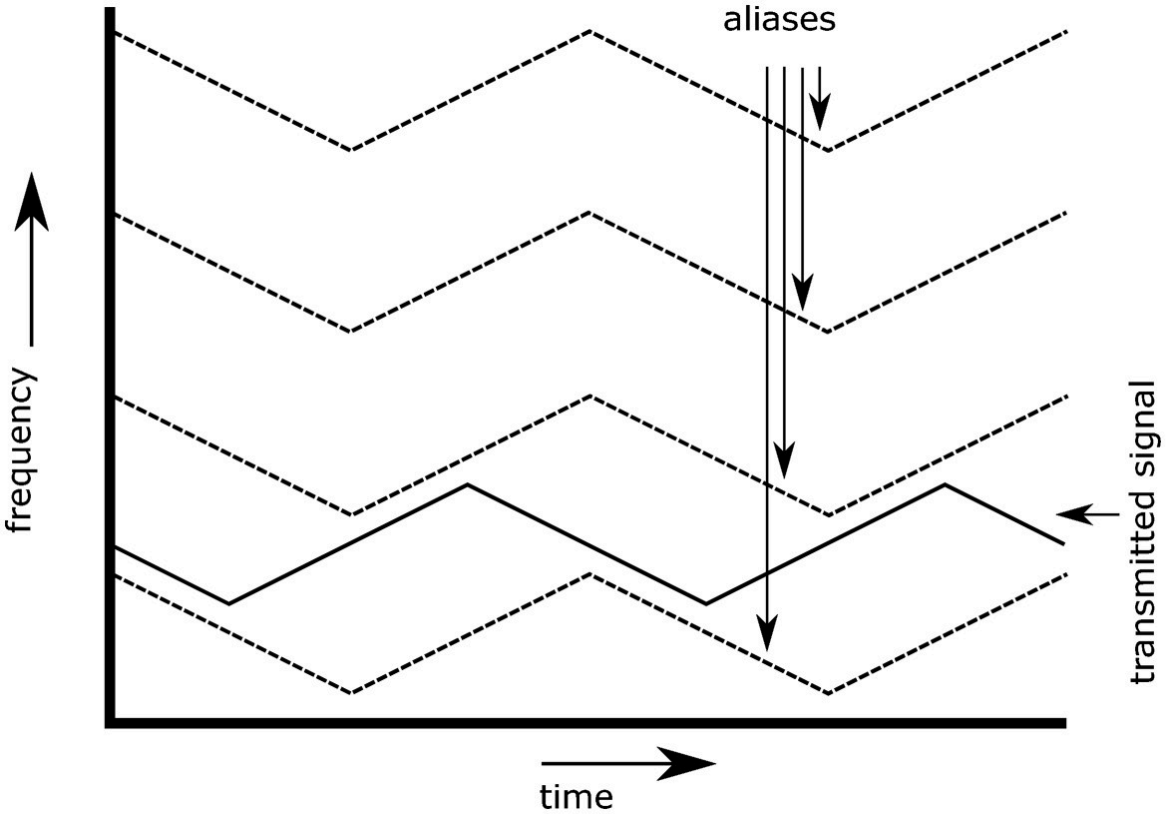
Aliases of an observed signal.

In the example of the FMCW radar, it means that the incoming signal can be observed with different frequencies. This means that multiple solutions are found when determining the Doppler shift of the incoming signal, and that the radial velocity of an object does not have a unique solution. In Fig 6, an observed signal is plotted in a dotted line, next to three of its aliases. As can be seen, it is not possible to determine which is the real signal and which are the aliases.

A solution to aliasing can be to increase the sampling frequency of the system. The difference between two aliases is equal to the sample frequency, so if this frequency is large, there is less possibility of signals being mistaken for one another. Another solution can be to decrease the center frequency of the transmitted signal, denoted as *f*_0_ in Eq 2. This means that for a given *V*_*R*_, the shift in frequency is also lower and it is less likely to get confused by aliases. Therefore the bandwidth of one alias is higher, and a higher value of *V*_*R*_ should occur in the test before the bandwidth is surpassed, as discussed in [16].

To completely prevent aliasing from existing, an infinite sampling rate is required. This is not possible, and a finite sampling rate will have to do. The sampling rate is limited by the quality and cost of available hardware, bearing in mind that the system is developed for use in GA and reductions in cost are desirable. Also, the center frequency used by the system is constrained, determined by bandwidth constraints by communication authorities. It is therefore not possible to completely prevent the occurrence of aliases in this hardware.

**Fig 7:**
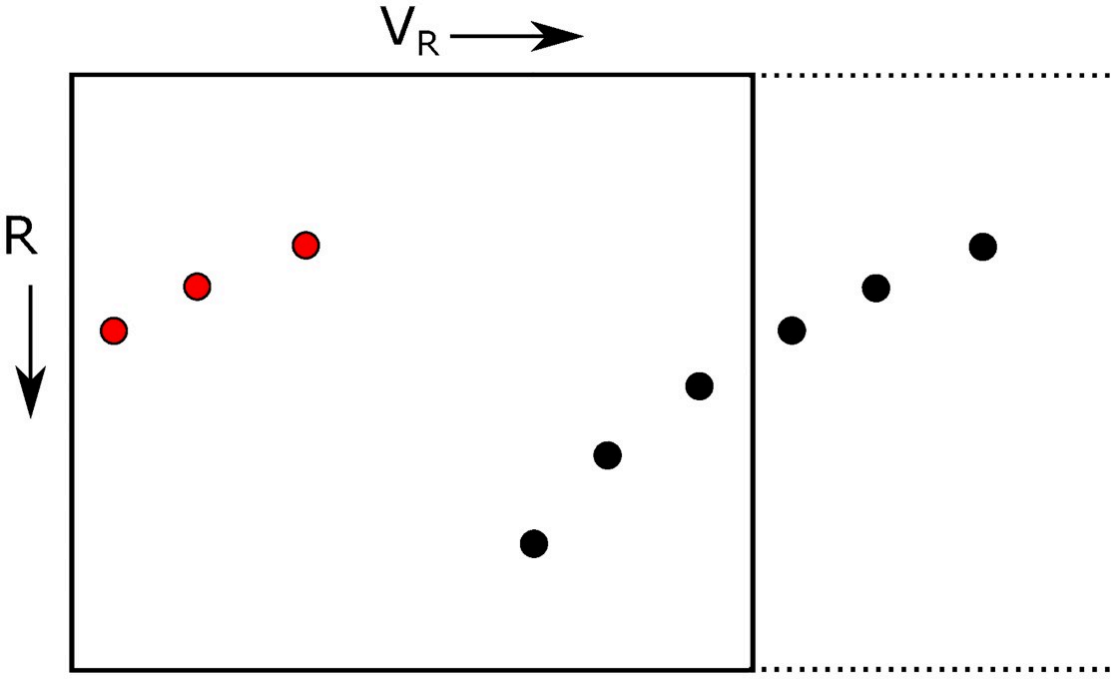
Aliases in the radar image.

In the radar image shown in Fig 4, aliasing will have as a consequence that the horizontal edges of the figure are adjacent to one another. This is illustrated in Fig 7, where the motion of an object is indicated in the radar image by a series of black dots. If the radial velocity of the object would increase to an amount that it would surpass the maximum limit of the horizontal axis, it would reappear on the other side of the image, as denoted by the red dots in the figure.

It is possible to solve the issue of aliasing. A solution is to use known information about the objects that are to be observed. It is also possible to use a series of observations in which the object is tracked over multiple time instances. If this is done, the change in range *R* can be used to validate the value of the radial velocity *V*_*R*_, as performed in [17].

As discussed above, the real signal and its alias cannot be distinguished from one another by instantaneous observation. Since the focus of this paper is on the detection of objects, it does not matter whether the original signal or one of its aliases is detected. In this work, aliasing is solved afterwards, by knowledge of the state of the other object, as will be discussed in section 4.

### 1.3 Steps required for sense-and-avoid

The radar image, as presented in Fig 4, is two-dimensional—with only one distance dimension. Aviation is three-dimensional, so more information is required to adequately notify the pilot of objects in the vicinity of the aircraft. Of course, one-dimensional safety measures do exist in aviation, such as vertical separation for air traffic and the TCAS-II [7, 18, 19]. It is possible to find more information about the state of any object that appears in the radar image.

Direction of Arrival determination algorithms exist, which can be used to determine the direction of an incoming signal. When this direction is known and the radar image provides the distance information *R*, the exact location of the other object can be found in three dimensions. Since the radial velocity *V*_*R*_ is measured, this can even give an indication of whether or not the other object is approaching the aircraft or not. To provide optimal information for the pilot, three-dimensional Direction of Arrival estimation is performed.

After the exact locations of objects are determined in one instance, a filter can be applied to remove reflections from the ground, such that only airborne objects and towers remain in the selection. The next step is to track the movement of the objects’ locations through time. This can happen either in the radar image, where the pixel needs to be tracked [17], or in three dimensional space, in such a way that the locations of the objects should first be determined before they are tracked.

When tracking is done, the next step is to predict the future track of the object. Predictions can be made based solely on the current state of the aircraft, extrapolating the current speed vector. The terrain around the aircraft may also be taken into account in the predictions of the other aircraft, as well as the local VFR flight routes. When predictions are made, conflicts between the own aircraft speed vector and the predicted other tracks can be detected. These conflicts may be presented to the pilot as is, or a conflict resolution advice may be included, assisting the pilot by providing a suggestion for safety.

- Data acquisition
- Radar image construction
- Pixel detection (This work, section 2)
- Direction of Arrival estimation (This work, section 3)
- Ground filtering
- Object tracking
- Conflict detection
- Resolution advice

## 2 Pixel detection

In this section it is described how the detection of objects of interest is performed in the radar image. In 2.1, it is discussed what an object is expected to look like in the radar image. The state of the art of existing object recognition software is discussed in section 2.2 and algorithms to perform corner detection are discussed in section 2.3. A first field test is performed, which is used to verify the expectations from section 2.1. The test is described in section 2.4. The presence of spurious signals, and the strategy to cope with them, is discussed in section 2.5.

### 2.1 Appearance of objects

It is important to consider what objects will look like when they appear in the radar image. In this section, the differences between radar images and optical images (pictures) are considered and described in detail.

#### 2.1.1 Different axes for images

Since the radar image has the axes of range and radial velocity, radar images are fundamentally different from visual images that we are accustomed to. The two axes of a picture indicate where the object was relative to the camera when the picture was taken. Objects that are closer appear bigger on the image. In the radar image, only one of the two axes relates to the position of an object. This is the range axis. This axis is also different from the two axes in pictures, which indicate the position of an object horizontally and vertically relative to the sensor.

The shape of the object will also differ in between the two images. In an optical image, a projection of the three-dimensional object is preserved, but this does not happen in the radar image.

#### 2.1.2 Mapping to the *R* and *V*_*R*_ axes

When a signal is sampled with a frequency of 10*MHz*, the distance that a radar signal travels between two samples is around 30*m*. This means that the range resolution of the radar image will be about 15*m* per pixel, taking into account that the signal needs to travel in two directions. This means that for most GA aircraft, all reflective parts of the hull will fall within the same range bin in the image, so the reflection will be displayed as a single pixel in range direction. For larger objects, the reflection may be seen in several range bins.

**Fig 8:**
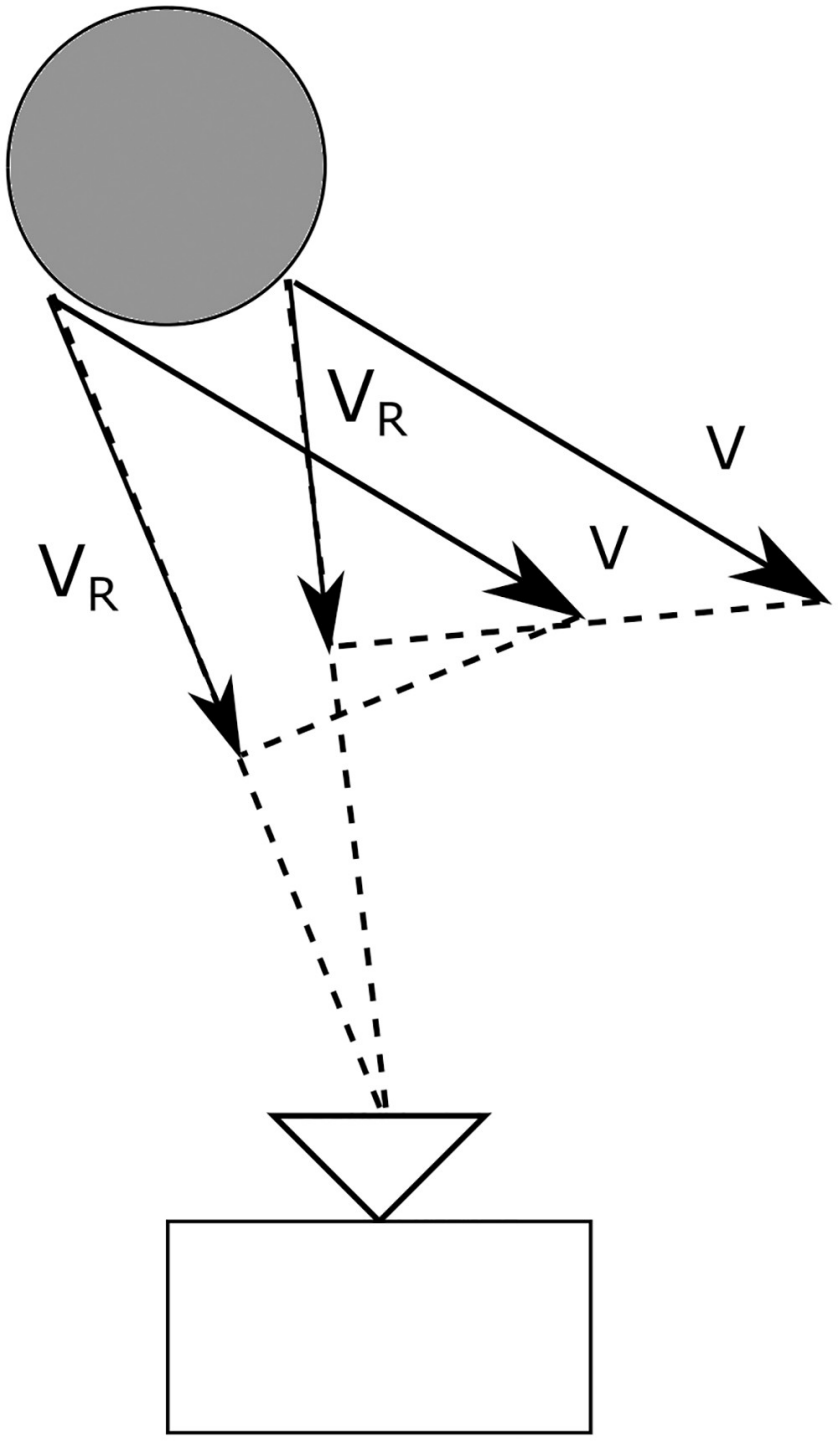
Two surfaces of an object close to the radar have a the same velocity $\vec{V}$ but different radial velocity *V*_*R*_.

For the velocity axis, it can be assumed that the entire object has the same velocity vector $\vec{V}$. Therefore the radial component of the velocity vector *V*_*R*_ will be equal for all reflecting surfaces, since the vector $\vec{R}$ is almost the same for the entire object. If the object comes close to the radar, small differences in *V*_*R*_ can be noticed as illustrated in Fig 8.

#### 2.1.3 Fourier analysis on a single signal

**Fig 9:**
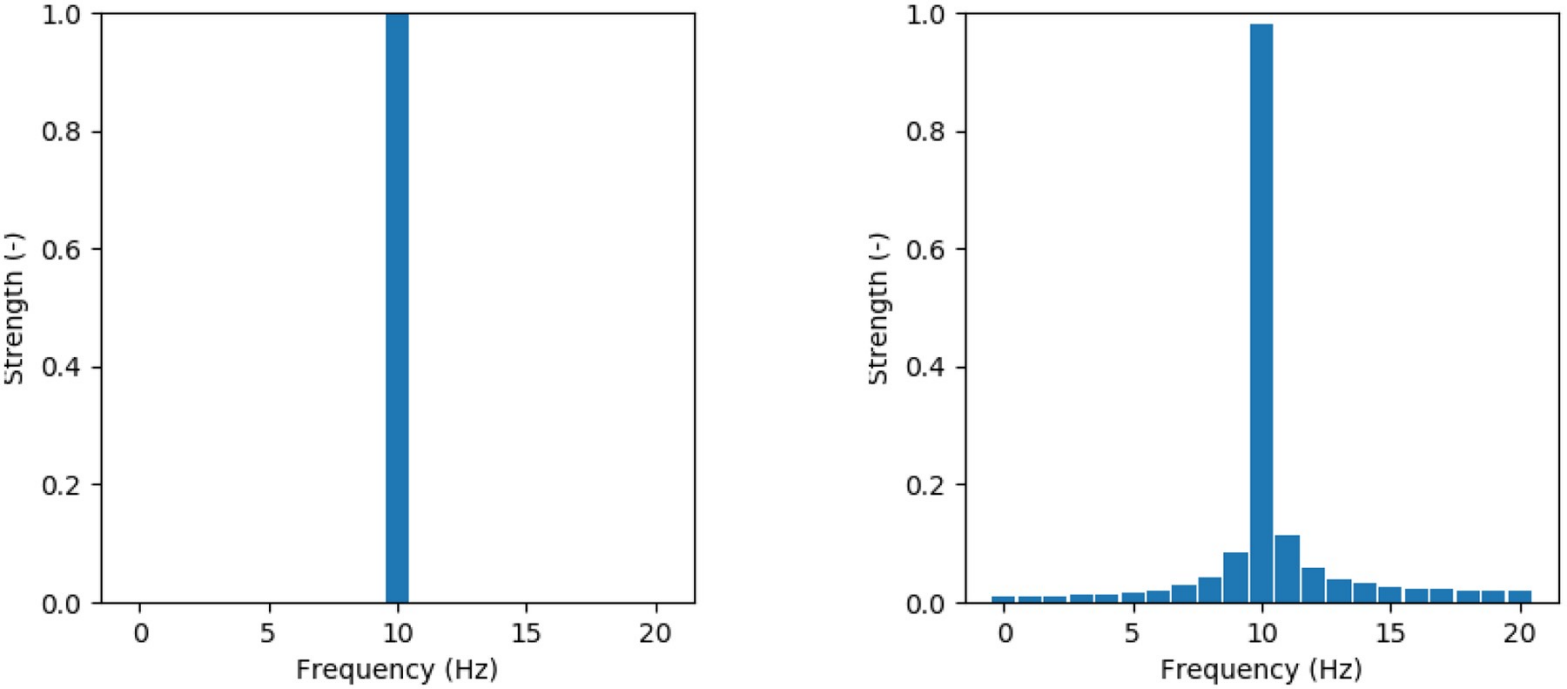
Bar graphs of one-dimensional Fourier results of two perfect sinusoids. A—*f* = 10 *Hz* B—*f* = 10.1 *Hz*.

Combining the results from the previous two paragraphs, it may be expected that an object is displayed in one single pixel in the radar image. It should be noted however, that the exact range and radial velocity of the object will not be the precise center values of the pixel in the radar image. The consequences of this are illustrated in Fig 9, where a one-dimensional Fourier analysis is performed on two sinusoids. The components of the two Fourier analyses are all integer frequencies. In Fig 9a, it is seen that the exact frequency of 10 *Hz*, is seen as one single bar, a one-dimensional pixel. However, in Fig 9b the frequency is not exactly the center frequency of a bin. So in this subfigure, the Fourier result is a sum of frequencies that lie around the original sinusoid of 10.1 *Hz*.

The effect illustrated in Fig 9 is representative for the effect in the radar image. Even though all reflective surfaces of an object may fall within the same *R*,*V*_*R*_ bin, the values will not be exactly the same as the center value of the pixel. Therefore, the Fourier analysis will yield a result where several nearby pixels are also illuminated. The pixel that the object falls in will still have the strongest signal.

The distance of the object will have an effect on two elements of its representation in the image. If the object is closer to the radar system, the distance is smaller and *R* will be smaller, so the location of the object will be more to the top of the image. Next to that, when an object is closer to the radar, its reflection will be stronger, so the pixels in the image will illuminate brighter.

#### 2.1.4 Resulting appearance in radar image

**Fig 10:**
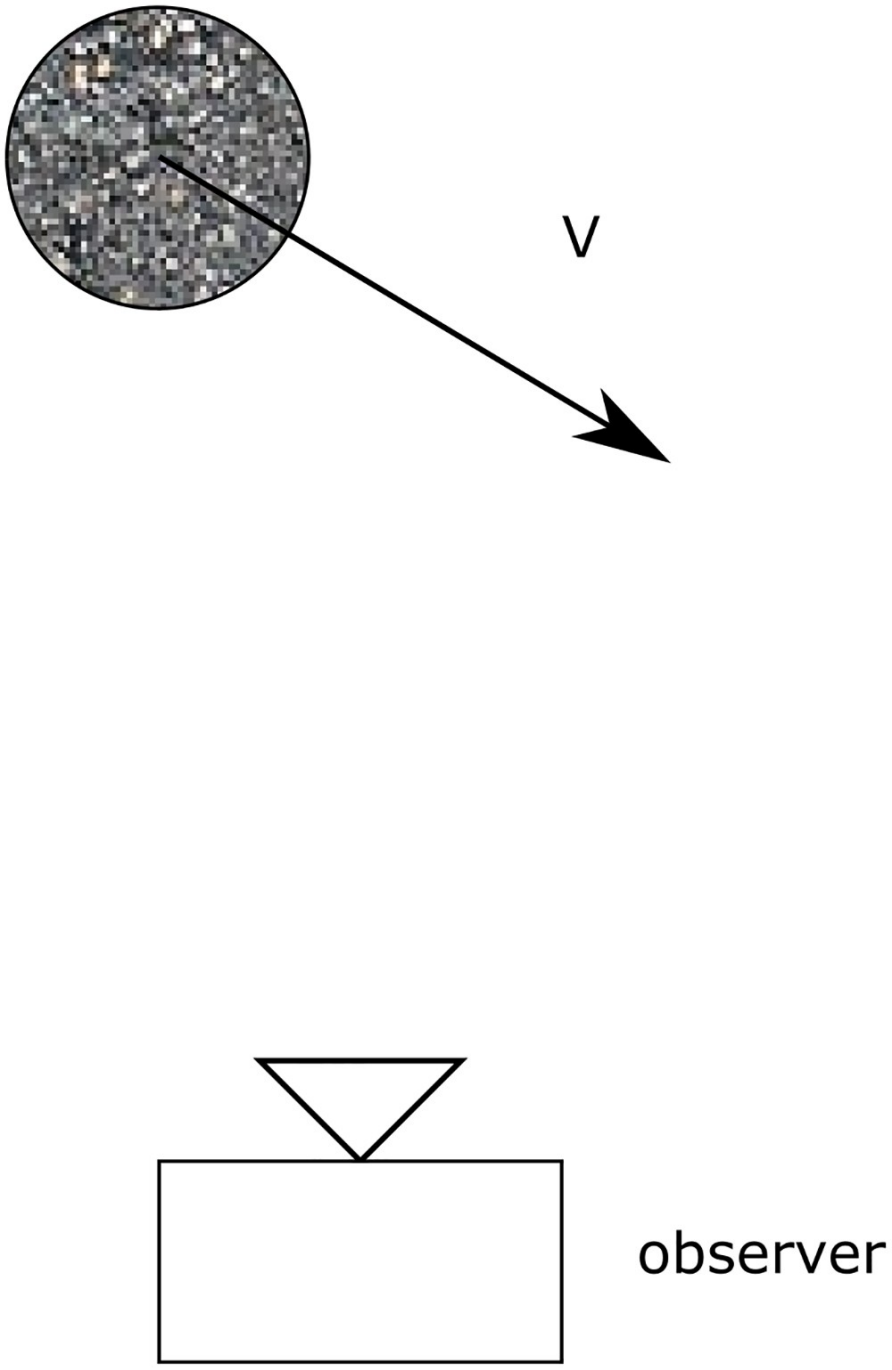
Top-down view of an object with speed vector $\vec{V}$ relative to an observer (camera or radar).

**Fig 11:**
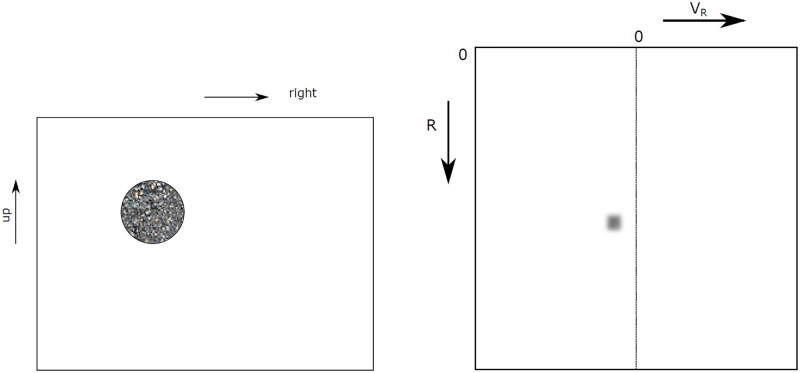
The object from Fig 10 as seen in a visual image and a radar image. A—Camera Image B—Radar Image.

Combining the considerations in section 2.1, it is possible to explain the appearance of objects in the radar image. In Fig 10, a situation is drawn where an object is being observed by a camera or a radar. In Fig 11, the resulting images of the camera and radar are drawn. It can be seen that in the optical image, the object is shown to the left, just as the situation in Fig 10. Also, the object has the same shape as the original. In Fig 11b, it is seen that the object is not seen as a round shape but as a small flock of illuminated pixels. Since the object is moving towards the sensor in Fig 10, the flock is located on the left side of the image, where *V*_*R*_ is negative.

### 2.2 Existing software

Even though the radar image is unlike an optical image, much research that is performed to pictures may be applicable to radar images. The radar image is still a two-dimensional figure, in which objects are to be found which have a higher intensity than the background. This compares to finding the bright spots in a grayscale picture. It is therefore possible to base the detection of objects on existing research on visual images.

Modern visual algorithms are capable of more sophisticated tasks than finding bright spots in a grayscale picture. Recent scientific papers deal with detection of continuously changing shapes in coloured videos [20]. Classification of any objects is also performed, categorizing the objects by the hand of their features. This can include noisy images with low resolutions, or moving cameras [21]. Face recognition is also performed by modern software [22].

This does not mean that finding objects in the radar image is trivial. The most straightforward strategy is to define a threshold above which a detection is concluded. Since objects that are closer reflect stronger than objects far away and objects are visible in several pixels in the radar image, this can have as a consequence that multiple pixels surpass the threshold, and that one object is detected as more than one. This can be solved by only using the highest value of the flock of pixels, but if two objects are quite close to each other in the radar image, they may be perceived as being one object.

### 2.3 Corner detection algorithms

The solution to the problem from section 2.2 is to use a Corner Detection Algorithm. Corners are defined as locations in the image that have diverging values with respect to their immediate neighbours, in both the horizontal and vertical directions. Various algorithms for corner detection exist. These algorithms vary in accuracy, consistency and speed. A Harris Corner Detection algorithm [23, 24] is well-known and widespread, and multiple researchers have found it to be an excellent algorithm [25, 26]. The Shi-Tomasi algorithm is a variation of the Harris algorithm, which makes the corner detection more suitable for tracking over time [27, 28]. Since the Shi-Tomasi corner detection algorithm can be found in the widespread OpenCV library, it is chosen to use it for this research.

The corner detector is public knowledge and widely available. Nevertheless, the core elements of the algorithm are presented below. The starting point is to compute the auto-correlation of the greyscale image, where the value of each pixel is compared to those in its immediate vicinity, as in Eq 3.

$$\begin{array}{c} E_{\Delta x,\Delta y}=\sum_{x,y}w_{x,y}{\left(I(x+\Delta x,y+\Delta y)-I(x,y)\right)}^{2} \end{array}$$ (3)

In Eq 3, the function *I*(.) denotes the intensity of the greyscale image in a specific pixel coordinate. The function *w*(.) is a windowing function with the output range between 0 and 1. Harris proposed to use a Gaussian smooth circular window, such that the response of the Corner Detection Algorithm would be invariable for rotation of the image.

Using a linear Taylor Series approximation and a linear matrix notation, Eq 3 is rewritten to contain a matrix *M*:

$$\begin{array}{c} E_{\Delta x,\Delta y}\approx \left[\Delta x\;\Delta y\right]\;\;M\;[\begin{array}{c} \Delta x\\ \Delta y \end{array}] \end{array}$$ (4)

In Eq 4, *M* equals:

$$\begin{array}{c} M=\sum_{x,y}w_{x,y}[\begin{array}{cc} I_{x}I_{x} & I_{x}I_{y}\\ I_{x}I_{y} & I_{y}I_{y} \end{array}] \end{array}$$ (5)

In Eq 5, the symbols *I*_*x*_ and *I*_*y*_ contain the image derivatives in *x* and *y* directions. As follows from Eqs 4 and 5, the covariance *E* of a single pixel is directly dependent on *M*, which is different for each pixel. *M* has the advantage that it is not dependent on the values of Δ*x* and Δ*y*, only on the local image derivatives *I*_*x*_ and *I*_*y*_ and the window function *w*.

The strategy from Shi and Tomasi is to compute the eigenvalues λ_1_ and λ_2_ of the matrix *M* for each pixel. If both eigenvalues are higher than a threshold value, the pixel is considered a corner.

### 2.4 First field test

**Fig 12:**
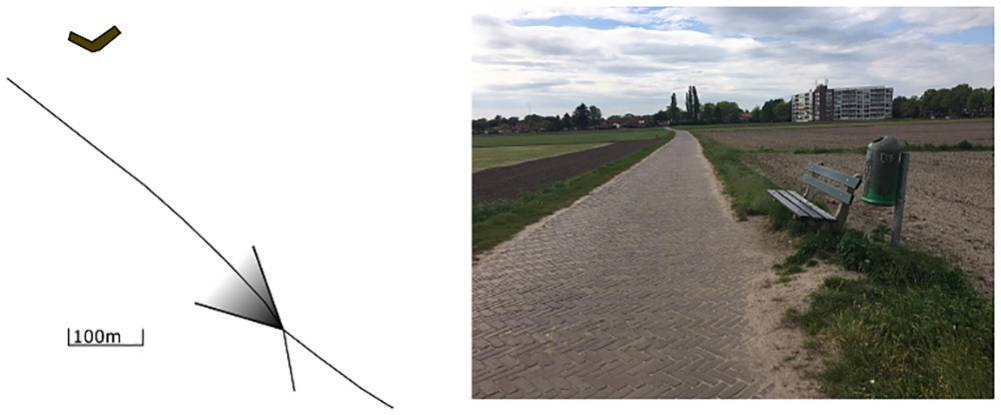
The location in Soest for the first field test. A—Local Map with roads, apartment building and the position of the radar indicated B—Picture with apartment building.

In order to prepare the experiments from section 4, a small field test is performed in which the radar was tested for the first time. This test takes place with a stationary radar on the ground. The location was in a meadow in Soest, in the Netherlands, with coordinates 52.172 degrees and 5.305 degrees for latitude and longitude. In Fig 12, the test location is illustrated by a marked map of the location and a picture.

In Fig 12a, the triangle indicates the position and looking direction of the radar, the circle is around a big apartment building that was clearly visible from the test location and the dashed line is a path used by cyclists and walkers. The path and the building are also seen in the picture in Fig 12b. This picture was not taken at the exact radar location but 150 meters forward. This was done in order to better show the apartment building, road and landscape in one image.

**Fig 13:**
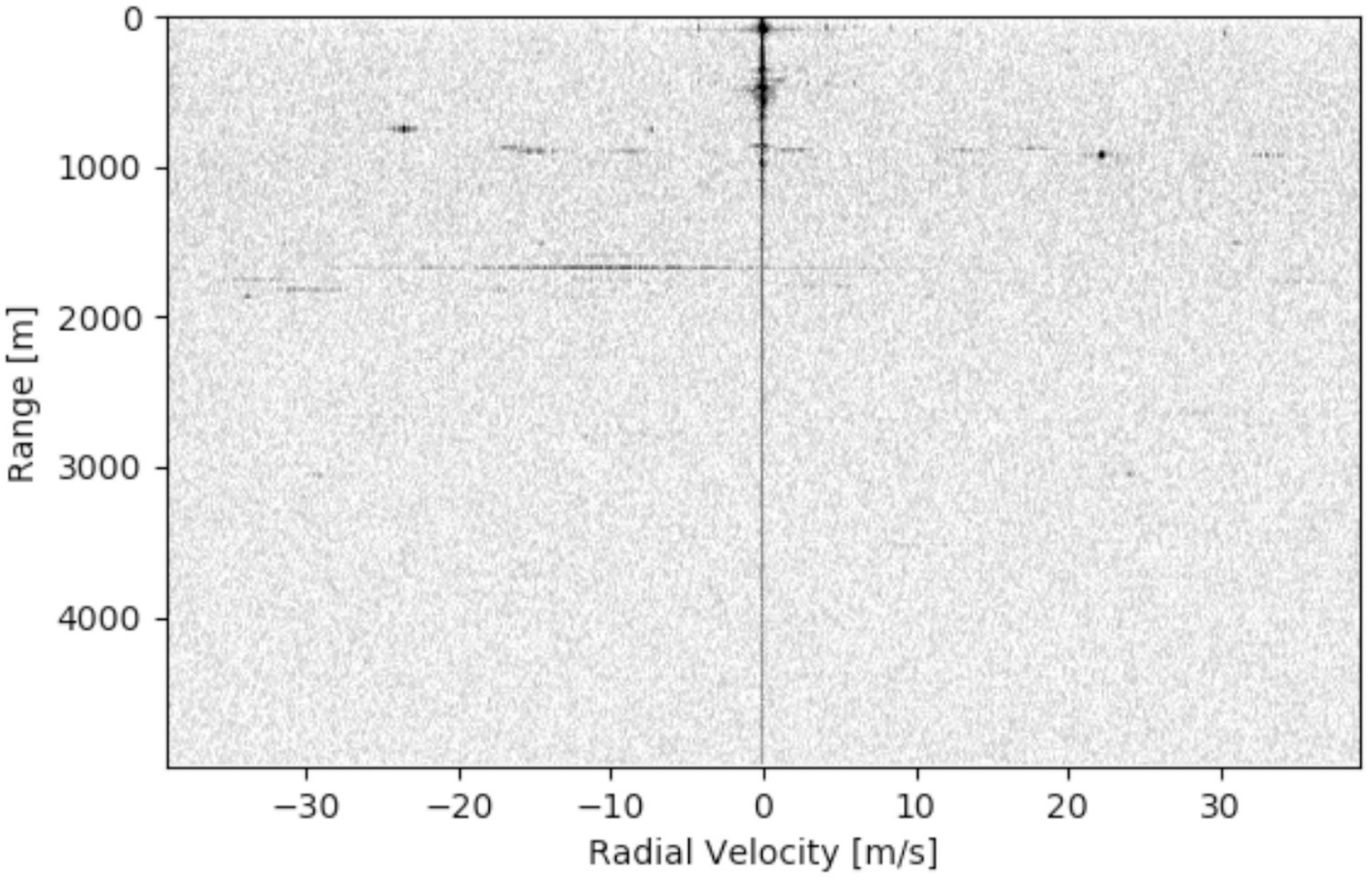
A snapshot from the results of the first field test (a darker pixel equals stronger reflection).

In Fig 13, a snapshot is shown from the field test in Soest. The axes of the image contain *R* and *V*_*R*_, as was described in section 1.1. In the image, brighter spots indicate strong signals and dark colours indicate that no signals are received with those values. It is seen that a vertical line is present in the image, indicating the line with *V*_*R*_ equal to zero. This is a often-seen consequence of Fourier analyses, where the zero-frequency component is offset with respect to the other frequencies. It can be seen that the strongest reflections are relatively close to *R* = 0. Several bright spots are observed around *R* = 950*m* and *R* = 1700*m*.

### 2.5 Removal of spurious signals

As seen in Fig 13, many illuminating spots can be seen in the radar image. However, this image was taking in stationary position with no moving objects nearby—no walkers or cyclists were present on the road. This raises questions about the reflections that are observed, particularly since they indicate movement with tens of meters per second. Moreover, the apartment building and the trees on the horizon were located around 450*m* from the radar and they blocked all objects behind them from view. The skies were partly overcast by clouds and no aircraft were observed—at least not by the eye. So it is remarkable that bright reflections are seen at 1000*m* and further. The spots do not move, even though the range should change if *V*_*R*_ ≠ 0, and the spots remain present if the radar is relocated.

These reflections are known as spurious signals, or spurs for short. They are consequences of imperfections in the radar hardware, such as interference between transmitter and receiver antennas [29]. Multiple strategies exist to cope with the existence of spurs, and different radar applications may require different solutions.

**Fig 14:**
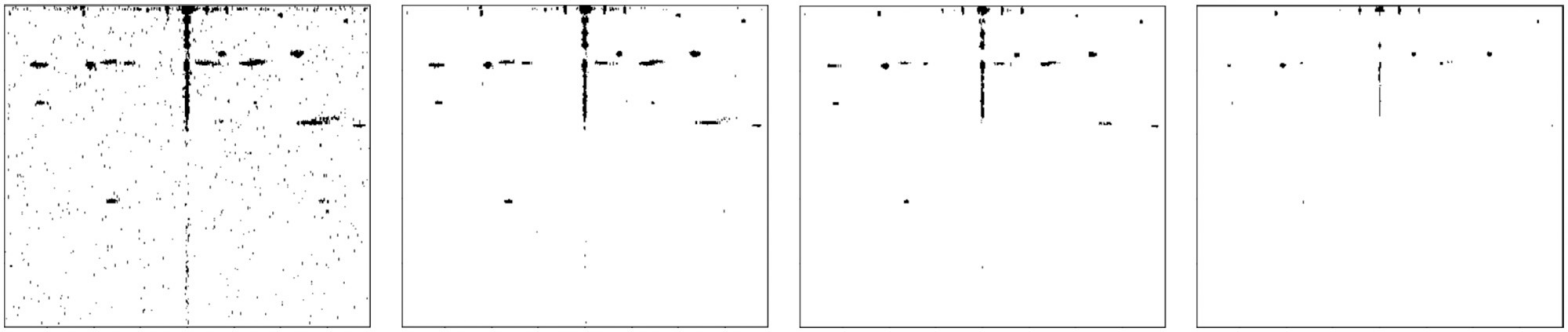
Spurs locations for different thresholds of minimum corner presence. A—>2% B—>5% C—>10% D—>25%.

Since the radar is being tested in a stationary position in a static environment, it can be concluded that all signals that are being observed now must be spurs. The challenge is to determine the locations of the spurs in the FMCW radar image. In order to do this, a series of frames are taken from the recorded data, and the Shi-Tomasi corner detector (section 2.3) is applied to find the locations of the corners. Because the corners tend to wiggle slightly, the corner locations are dilated, such that the adjacent pixels are also counted as spurs. From a series of binary corner images, it is computed how often a pixel is detected as a corner. Pixel counts that surpass a threshold are considered spurs. The results of different thresholds are seen in Fig 14.

The use of the spurs image is that now the locations of the spurs in the radar output are known. If the Shi-Tomasi corner detection algorithm finds corners that lie within the spurs on the map, these corners are disregarded for further investigation.

## 3 Direction of arrival

In chapter 2, it was explained how the radar system can detect objects of interest in the radar image. This strategy can provide a user with Range and Doppler information about airborne objects, but this information is not sufficient to tell the user where a hazard is coming from. Additional steps are therefore required to improve the situation awareness of the radar user. The goal of this is to determine the Direction of Arrival (DoA) of an incoming radar signal. If this is possible, the information can be combined with the Range information to pinpoint the location of the object.

In this chapter, the technique for DoA estimation is explained. In section 3.1 different strategies to perform DoA estimation are introduced, and it is explained which one is chosen in this project. The next section, 3.2, contains the algorithm that is used to perform DoA in three dimensions. Calibration of the experimental setup is required in order to achieve accurate results, this is explained in section 3.3. The last section, 3.4, contains the results of the DoA estimation of the first field test, which was introduced in section 2.4.

### 3.1 DoA by phase difference

Several different techniques exist for determining location information for radar. Airport Surveillance Radars, used by Air Traffic Control, only provide 2D information. For airports, this is solved by using a Secondary Surveillance Radar [30], which interrogates the transponder on board of the aircraft for altitude information. This solution is not suitable for this GA application, as an SSR is unsuitable for taking on board of an aircraft and not all objects can be expected to be equipped with the proper transponders.

Another solution to determine an object’s location is to perform triangulation with multiple sensors that measure distance independently. For this strategy, the distance between the sensors and the accuracy of the range measurements determine the quality of the results. For GA aircraft, multiple sensors could be at most about 10 meters apart from each other, but for the hardware, the range resolution is not expected to become smaller than 5*m*, so this would leave a very poor directional estimate.

Directional antennas can also help in localizing an object. The principle of those is that a directional antenna broadcasts a signal in a single direction, so any return signals that are observed must originate from that direction. Examples of these are the Primary Radar itself, or Height Finding Radars [31] which are directional because a parabolic reflector is built around the antenna. Phased arrays [10, 32] can also be a solution for transmitting a directional signal, by having multiple transmitters next to each other in parallel. The problem with directional antennas is that only one direction can be observed in a single moment. In order to observe the entire space around the aircraft, a scanning pattern is needed, in which the size of the beam, the total coverage and the scanning speed must be balanced to each other.

**Fig 15:**
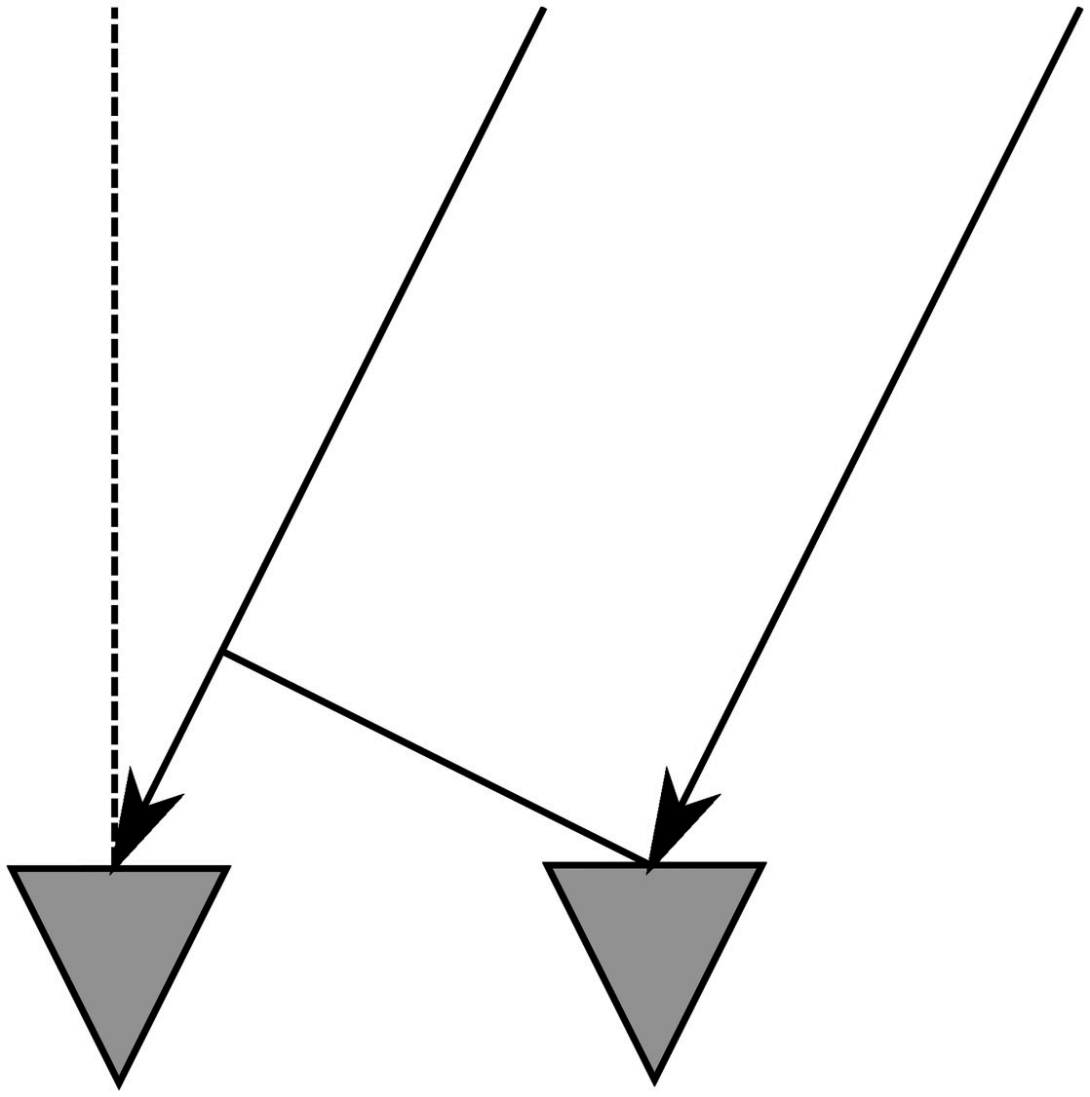
Phased array principle: A signal arrives with an angle and is received later by the left antenna.

The chosen solution is to compute the DoA by the hand of the phase difference of multiple adjacent antennas, as illustrated in Fig 15. This strategy is similar to using a phased array, but the direction is computed when the signals are received, and not predetermined when they are transmitted. A disadvantage of this is that each received antenna needs to be recorded separately, instead of a simple addition of all incoming signals as is seen in a phased array. The positive side is that the hardware can receive incoming signals from many directions, and therefore large parts of the close environment can be observed.

### 3.2 Three-dimensional algorithm

**Fig 16:**
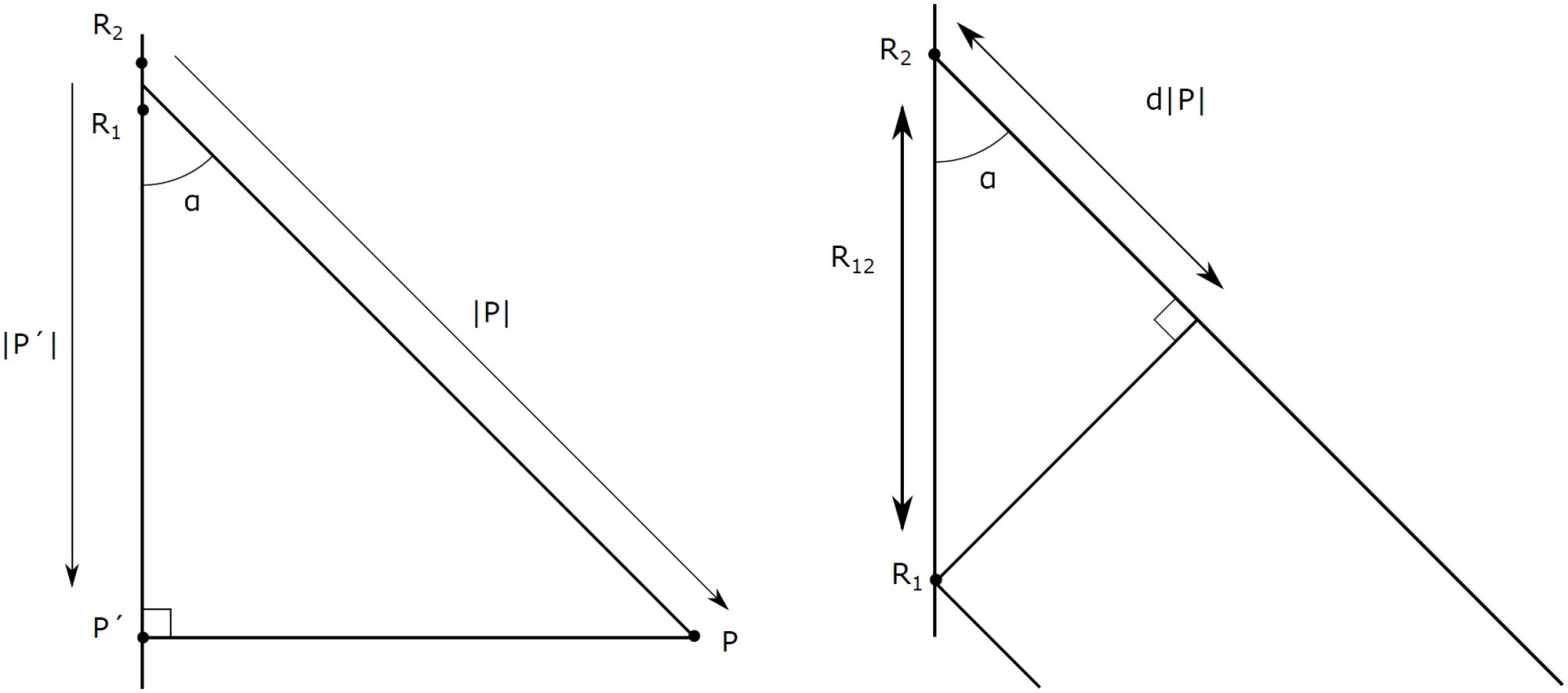
Definitions of incoming signals on two antennas *R*_1_ and *R*_2_.

In this section the algorithm is presented to determine an object’s location in three dimensions, when the signals are received by multiple receivers *R*_*x*_. In Fig 16 the geometry of the situation is illustrated. The location of the point *P* is denoted as the vector $\vec{P}$, as to avoid confusion with the receivers *R*_*x*_.

When the distance of $\vec{R_{12}}$ is small with respect to $\vec{P}$, angle *α* is identical in Fig 16a and 16b, so the triangles indicated in these figures must be similar, meaning that the following relation holds:

$$\begin{array}{c} \frac{|{\vec{P}}^{\prime }|}{|\vec{P}|}=\frac{d|\vec{P}|}{|\vec{R_{12}}|} \end{array}$$ (6)

The phase difference *ψ*_12_ between the antennas *R*_1_ and *R*_2_ is directly dependent on the distance difference $d|\vec{P}|$ and the wavelength λ of the carrier frequency. Eq 6 is rewritten and afterwards rearranged:

$$\begin{array}{c} \frac{|{\vec{P}}^{\prime }|}{|\vec{P}|}=\frac{\psi_{12}\lambda }{2\pi }\cdot \frac{1}{|\vec{R_{12}}|} \end{array}$$ (7)

This leaves the rather obscure term $|{\vec{P}}^{\prime }|$ in the equation: this is the distance to the point *P*′. That point is found when *P* is projected on the line $\vec{R_{12}}$. This needs to be removed from the equation, and therefore $|{\vec{P}}^{\prime }|$ is written as the product of the vector $\vec{P}$ and the unit vector in the direction from *R*_1_ to *R*_2_.

$$\begin{array}{c} |{\vec{P}}^{\prime }|=\frac{\vec{P}\cdot {\vec{R}}_{12}}{|{\vec{R}}_{12}|} \end{array}$$ (8)

Inserting Eq 8 in Eq 9 yields to the relation between the phase difference *ψ*_12_, the antenna distance *R*_12_ and the source position *P*:

$$\begin{array}{c} \frac{{\vec{R}}_{12}}{|{\vec{R}}_{12}|}\cdot \vec{P}=\frac{\psi_{12}\cdot \lambda }{2\pi }\frac{|\vec{P}|}{|\vec{R_{12}}|} \end{array}$$ (9)

From this equation, the term $|{\vec{R}}_{12}|$ falls away on both sides. This equation can be applied to any combination of two receiving antennas, *R*_*x*_, as long as the phase difference between the two is measured and their positions are known:

$$\begin{array}{c} \vec{R_{A}}\cdot \vec{P}=\frac{\psi_{A}\cdot \lambda }{2\pi }\left|\vec{P}\right|\\ \vec{R_{B}}\cdot \vec{P}=\frac{\psi_{B}\cdot \lambda }{2\pi }\left|\vec{P}\right|\\ \vec{R_{C}}\cdot \vec{P}=\frac{\psi_{C}\cdot \lambda }{2\pi }\left|\vec{P}\right|\\ \ldots \end{array}$$ (10)

These equations can be found for all combinations of *R*_*x*_ that receive the signal, and put into matrix form:

$$\begin{array}{c} \frac{2\pi }{\lambda }[\begin{array}{c} \vec{R_{A}}\\ \vec{R_{B}}\\ \vec{R_{C}}\\ \ldots \end{array}]\cdot \vec{P}=\left|\vec{P}\right|[\begin{array}{c} \psi_{A}\\ \psi_{B}\\ \psi_{C}\\ \ldots \end{array}] \end{array}$$ (11)

When Eq 11 is constructed, all known parameters are sorted on the left side, since the carrier frequency λ is constant and the vectors $\vec{R}$ depend on the antenna geometry. On the right hand side, all measured parameters are placed. This includes $|\vec{P}|$, since this is the range to the object, which is measured directly by the FMCW frequency delay (explained in chapter 1). This means that the equation is now written in the form $a\cdot \vec{x}=\vec{y}$, meaning that the equation can now be solved as a linear least squares problem, and $\vec{P}$ can be computed. This is the location of the source *P* of the reflection of the radar signal.

However, the linear least squares can only give a location $\vec{P}$ that lies in the span of the vector space of *R*. That means that if the antenna locations are spread in three dimensions, then the location of $\vec{P}$ can also be found in *R*^3^ (assuming that $\vec{P}$ is in view of the antennas). If all antennas are placed in a horizontal line, the location of $\vec{P}$ can only be determined in horizontal direction. And if all antennas are placed in a plane, then only the projection of $\vec{P}$ on that plane can be found.

**Fig 17:**
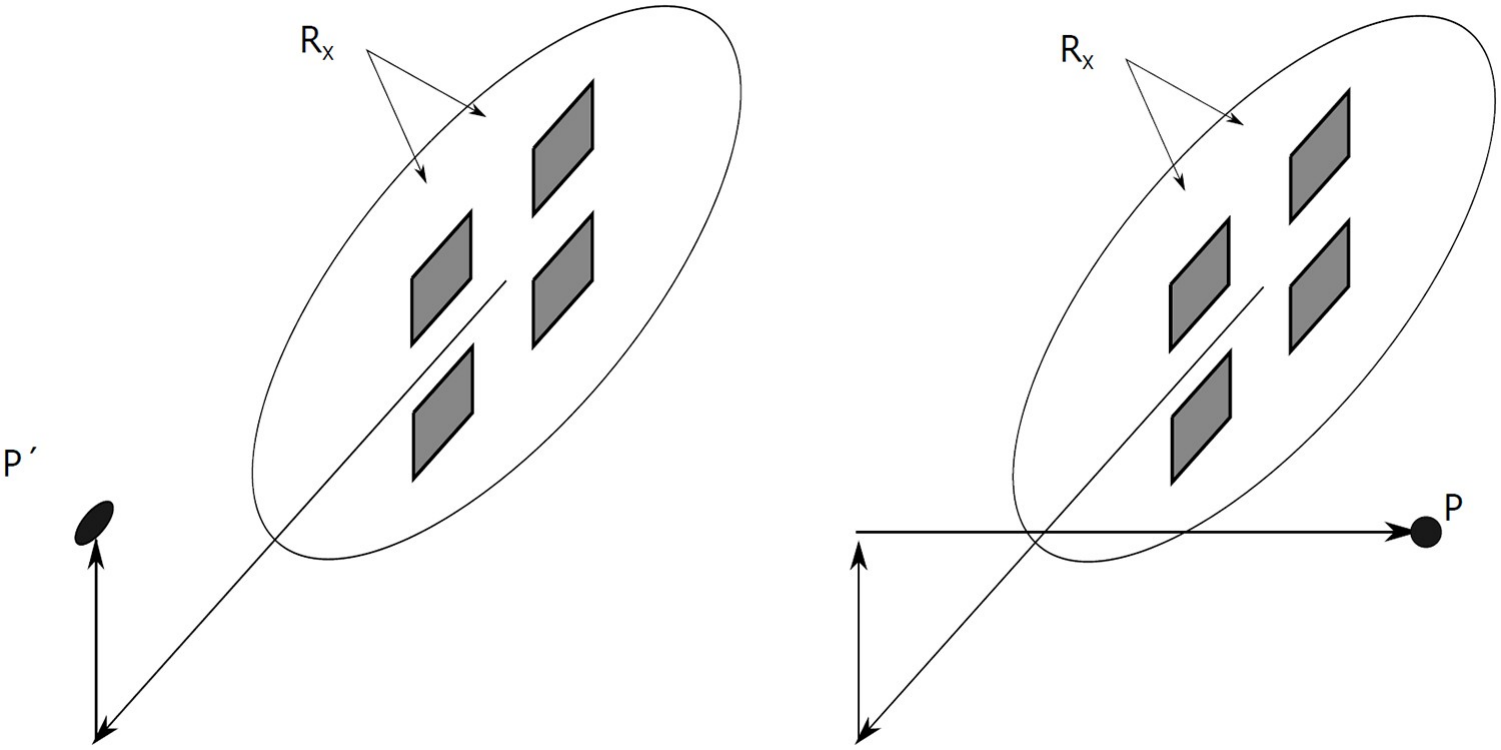
Results of 3d DoA with a radar with 4 coplanar receiving antennas.

For the FMCW radar that is used in this research, a constellation of four antennas is used that are located in a plane. This means that an extra step is required to determine an object’s location in *R*^3^. Luckily, this is possible. Since the total distance to the object is known and two coordinates span the plane dimensions, the Pythagorean theorem can be used to determine the perpendicular distance. This extra step is illustrated in Fig 17.

### 3.3 Antenna calibration for imperfect phased array

The strategy described in section 3.2 works very well if the assumptions from Fig 15 can hold perfectly. However, in reality measurements are often distorted by imperfections in the equipment. This also applies for the radar equipment, which needs to be calibrated before DoA estimation can be performed accurately. The reason for this can be the presence of small dirt particles on the sensors, or minor differences in the lengths of the antenna cables to the analog-to-digital converter. This means that in practice, noise terms *n* should be removed from the measured phases *ψ*_*x*_ in Eq 11:

$$\begin{array}{c} \frac{2\pi }{\lambda }[\begin{array}{c} \vec{R_{A}}\\ \vec{R_{B}}\\ \vec{R_{C}}\\ \ldots \end{array}]\cdot \vec{P}=\left|\vec{P}\right|[\begin{array}{c} \psi_{A}-n_{A}\\ \psi_{B}-n_{B}\\ \psi_{C}-n_{C}\\ \ldots \end{array}] \end{array}$$ (12)

Note that in Eq 12 the terms *n* may be positive or negative and are unknown by the design. In order to be able to use the equation, the terms *n* must be found by calibration of the hardware.

Several methods have been developed to find the antenna noise, which not only consists of a phase delay but also of an amplitude error [32–34]. These algorithms perform calibration to a point or object, of which the position is known. For the first field test described in section 2.4, this can be done.

### 3.4 Results for first field test

**Fig 18:**
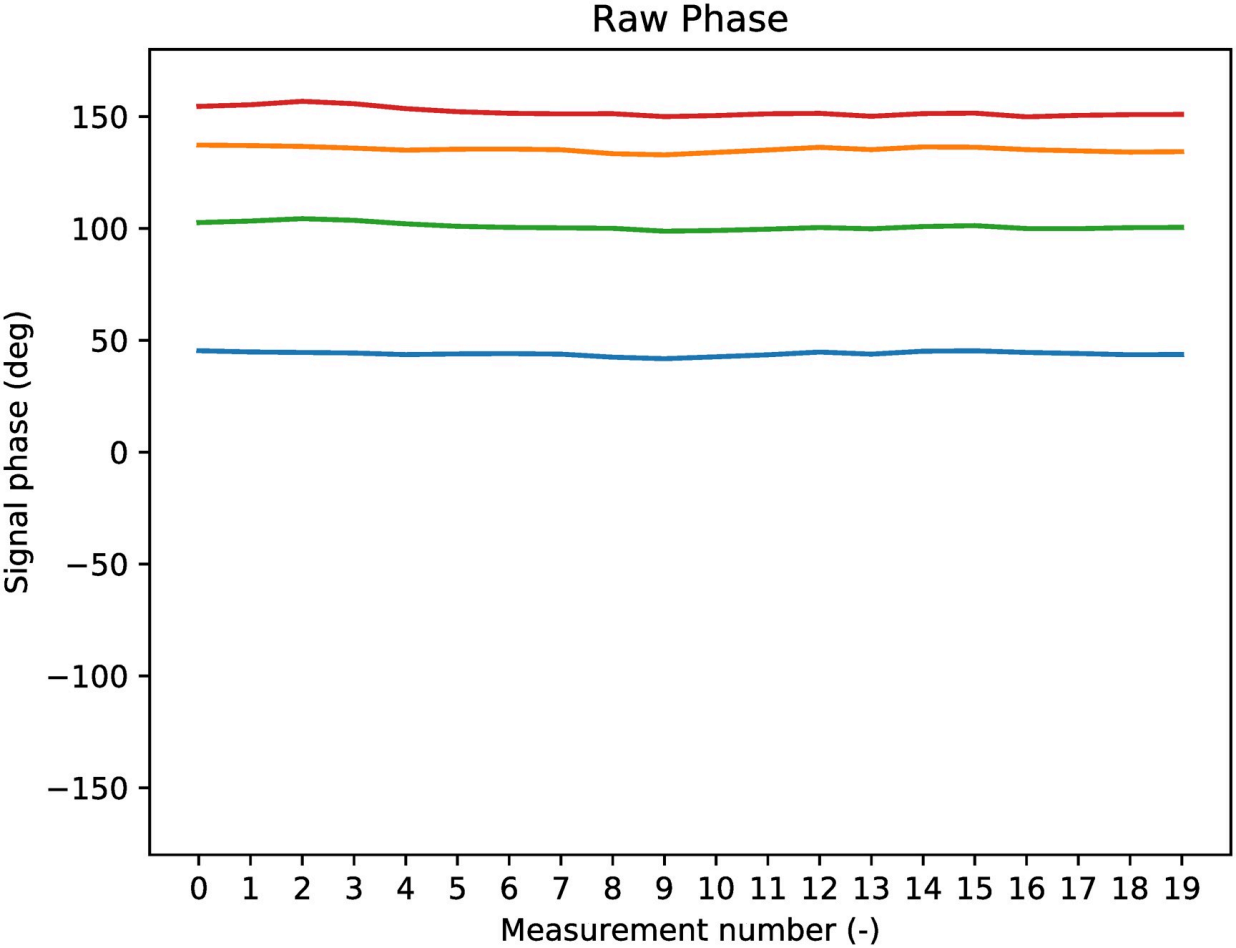
Raw signal phase of target reflection.

In this section, the results of DoA estimation after calibration of the radar are presented. For the first field test from section 2.4, A measurement on the satellite map indicated that the distance to the apartment building from Fig 12b was about 438 meters away from the measurement location. Indeed, a strong reflection was seen by the radar in the Fourier bin 430*m* − 450*m*, with velocity 0*m*/*s*—as can be seen in Fig 13. The raw phase of the measured signals is plotted in Fig 18, where it can be observed that the measured phase is relatively constant over a period of 20 measurements.

**Fig 19:**
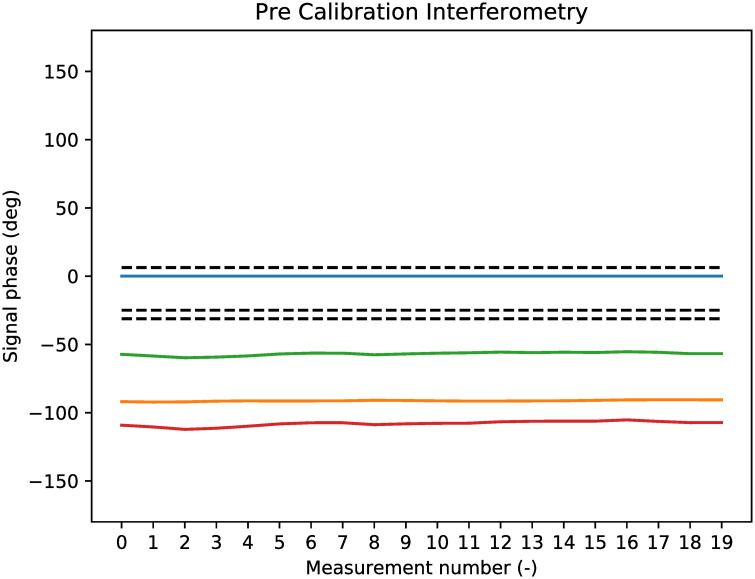
Antenna phase results of target reflection before calibration.

Since the phase of the raw signal is constant, the phase difference between the antennas is also constant, as seen in Fig 19. In this figure, the phase difference is shown for Antenna 1, compared to all other antennas. Antenna 1 is the blue line, therefore the blue line is always at 0. The phase difference that is theoretically expected by the measurements is also indicated in the figure: these are the black dashed lines. Since the position of the apartment building at the field test was not directly in front of the radar (but 5 degrees to the right of and 1 degree below the radar central axis), the expected phase differences are not zero. The expected phase difference is computed with the geometry from Fig 16, since the direction of *P* is known.

**Fig 20:**
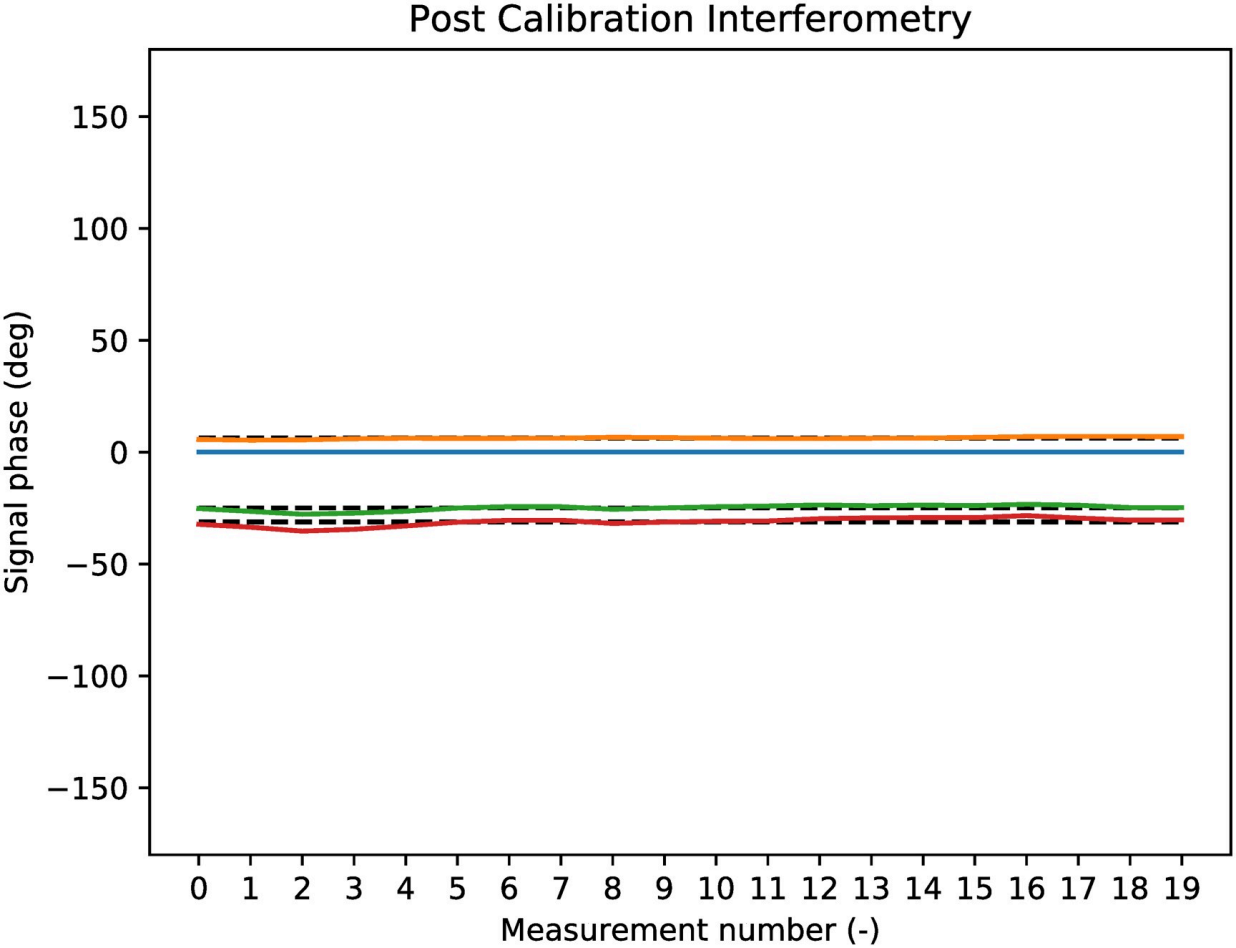
Antenna phase results of target reflection after calibration.

The algorithm from [33] is used to compute the phase difference that is required to align all coloured lines in Fig 19 with the black dashed lines for the expectations. In other words, the algorithm computes the terms *n*_*x*_ from Eq 12. When the noise terms *n*_*x*_ are known, they are removed from the raw signals and the image for the phase difference is made again, as found in Fig 20. In this figure it is seen that the phase differences are now close to the expected black dashed lines. In fact, the average offset of a phase difference and its theoretically expected value is now 0.9*degrees*.

**Fig 21:**
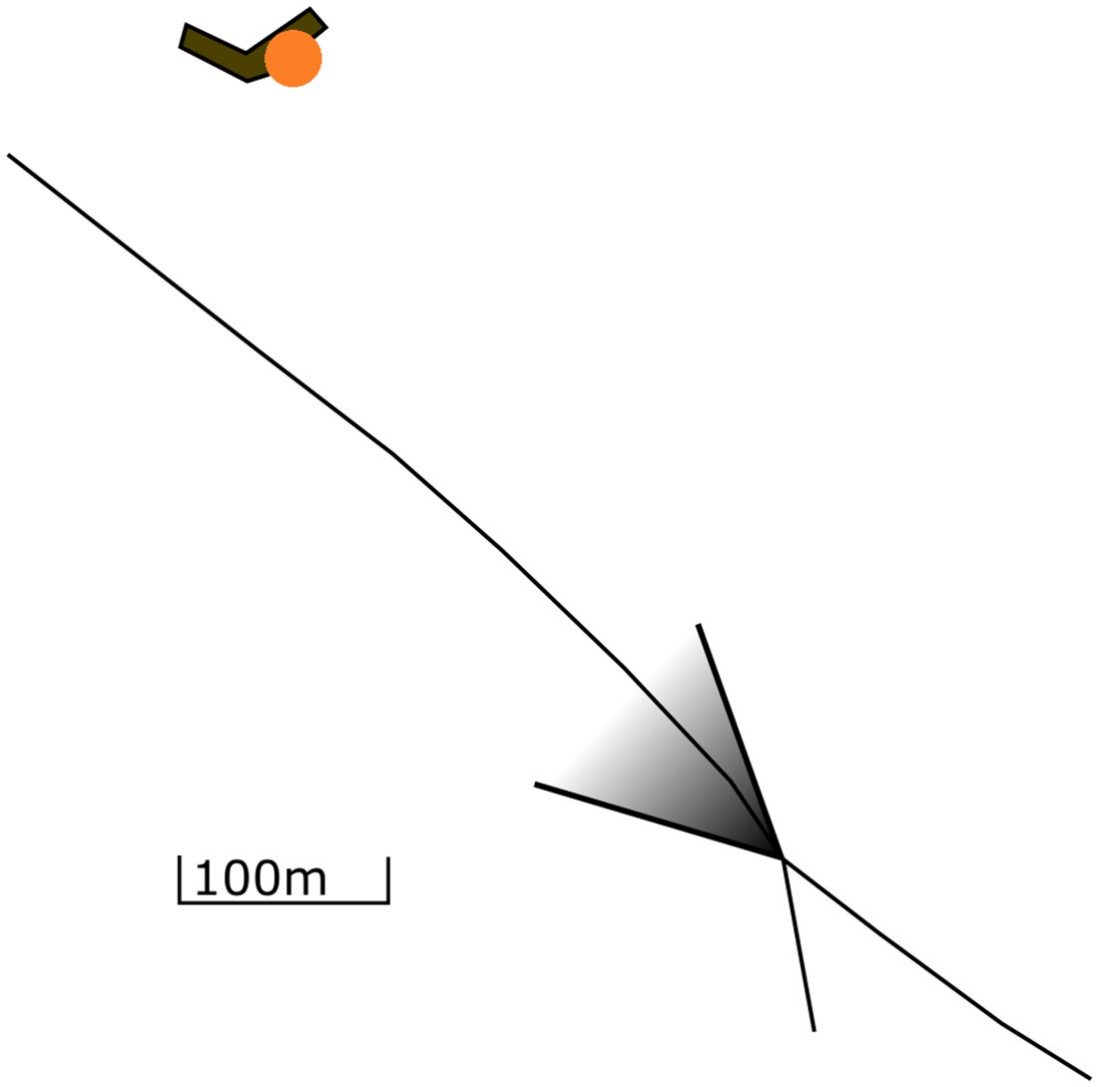
Location of the scatter after calibration and DoA estimation, indicated on the map from Soest.

Now that the calibration is done, it is possible to determine the location of the apartment building in 3D with the formula from Eq 12. When taking into account the attitude of the radar setup, the reflection is found to be 372*m* to the north, and 239*m* to the west. Also, the reflection is coming from 16*m* above the measurement station. This can be explained since the apartment building is 7 stories high. When put on the map from image Fig 12a, the location of the building is indicated with a dot. The result can be seen in Fig 21.

The location of the reflection is determined for all time instances in the first field test, and the results are always similar to Fig 21, with the location of the reflection determined on spot of the apartment building. It was already expected that the location of the reflection would be found at the front of the building, since the 3D results were calibrated to the location of the building. It can be concluded that the calibration algorithm works correctly and that the results are consistent.

## 4 Experiment

In this section, the flight experiment is described which is done in order to assess the performance of the radar hardware. The type of the aircraft, flight information and dependent variables are discussed in sections 4.1 to 4.3.

### 4.1 Aircraft type

**Fig 22:**
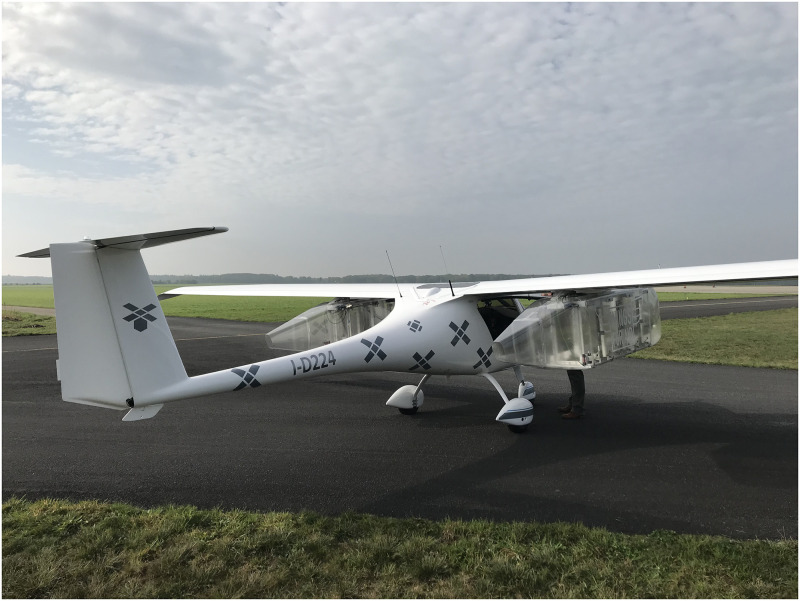
The aircraft used for detection, with the cargo boxes under the wings.

The aircraft used in the flight experiment is of the type Pipistrel Virus 912. These aircraft belong to the category ultralight, with a fuselage that is as small as possible, suitable for only two pilots. An image of the aircraft can be seen in Fig 22. The aircraft is about 6*m* long with a wingspan of 12*m*. Since the aircraft is small, the surface for radar reflections is also small and aircraft of this type are expected to be amongst the most difficult GA aircraft to detect with the radar.

The aircraft is equipped with two external freight boxes that were carried under the wings. These happened to be present for other purposes other than this experiment, but they do have an influence on the test results since they will increase the radar cross-section of the aircraft, causing it to reflect more signals and therefore to be easier to be seen. The effects of the boxes have not been quantified in this study.

The aircraft is also equipped with a GPS tracker. In this way, the position of the aircraft is known at all times, and the results of the DoA estimation can therefore be compared to the actual location of the aircraft. This is done after the experiment, when the DoA results have been computed.

### 4.2 Flight information

The experiment is performed at the area of Deelen Air Base in the Netherlands, of which the airspace was closed off for traffic other than the experiment aircraft. Any airborne reflections must have come from the test aircraft or from birds that happened to be in the air. No birds were observed with the eye during the experiment.

**Fig 23:**
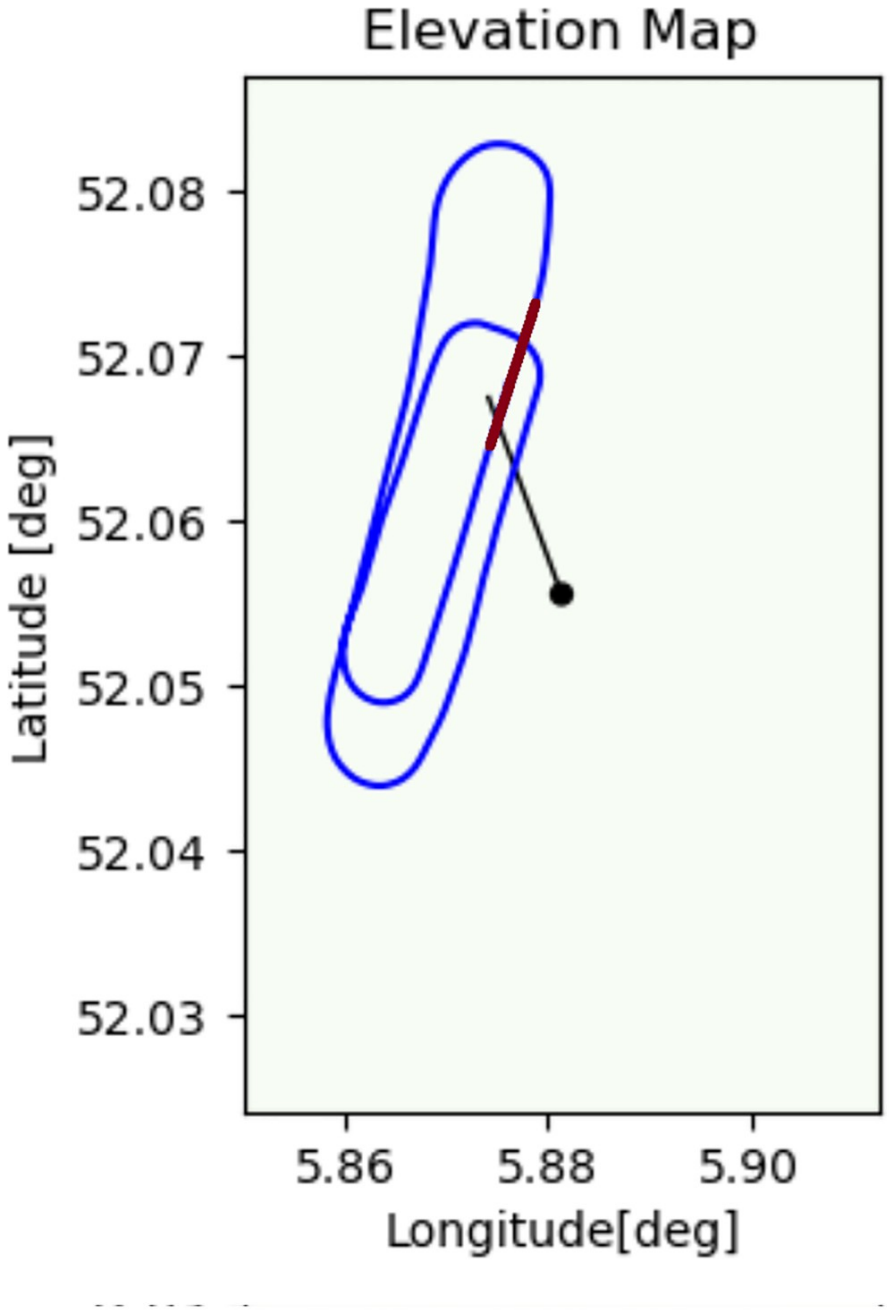
Ground track of the flight in Deelen. The radar location and looking direction are indicated in black, the red line shows the location of the runway. The flight was in counterclockwise direction.

The aircraft took off from the runway and flew one complete circuit over the field before finishing the route to go for landing. The radar was located at a small hill -large enough to arise above the tall grass, stationary aimed towards the sky above the runway. During the flight, the aircraft was always between about 500*m* and 3000*m* distance from the radar. The ground track of the flight can be seen in Fig 23.

### 4.3 Dependent variables

As dependent variables for the experiment, the differences between the recorded and observed positions of the aircraft are used. First, the range and radial velocity are computed at the hand of the GPS results and compared to the measured values. Secondly, the location of the aircraft is expressed in Cartesian coordinates in the radar centred axis system. The absolute difference of the found and tracked locations is computed. In order to evaluate the performance of the DoA algorithm, the offset will also be expressed in azimuth and elevation as seen from the radar. The last step to be taken is to apply a simple low-pass filter on the Cartesian results, in order to tackle the presence of high-frequency noise.

## 5 Results

**Fig 24:**
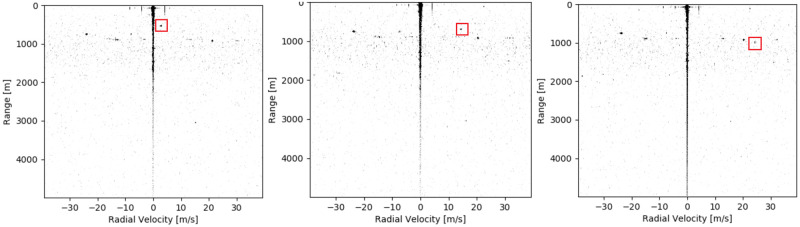
Three screenshots of the radar output during the flight over the radar. The aircraft reflection is indicated with a red square.

In this section, the results of the experiment are presented and described. The results are discussed briefly in this chapter; a more elaborate discussion can be found in section 6. First of all, the resulting radar output is shown. In Fig 24, three snapshots are shown of the radar images during the flyover of the aircraft. It can be seen that the images are very similar, but a moving cluster of pixels is observed. This is the reflection of the aircraft, passing over the airfield.

**Fig 25:**
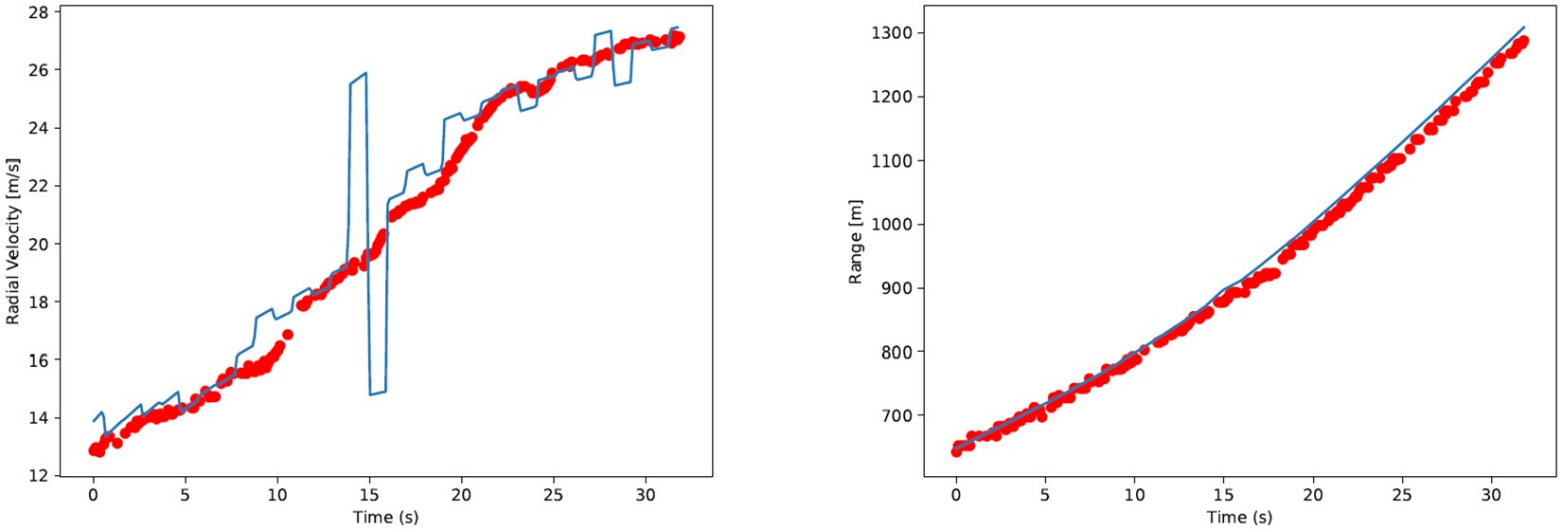
Results for *V*_*R*_ and *R* during the flyover in the experiment for radar (red dots) and GPS (blue line) measurements.

The pixel detection alogirhtm (including the spurs filter) is applied to the radar images, and resulting coordinates of the tracked pixel are plotted. Two plots are made, for the Range and Radial Velocity, which contain the measurements from both the radar and the GPS. The GPS does not yield the results for *V*_*R*_ directly, but they can be simply computed since the position of the radar is known, since this means that the distance vector from radar to aircraft is known. The results of these are seen in Fig 25.

In Fig 25, it is seen that the results for the radar resemble the results from the GPS, where the radial velocity and range start small in the experiment and gradually rise to higher values. The shape of the curve is also similar, but it should be noted that the results of the GPS vary a lot when determining the radial velocity. Also pay attention that not for every measurement a red dot is plotted: sometimes the pixel detection algorithm found that the radar feedback was not strong enough to pass the detection threshold. In the 300 measurements in 32 seconds during the flyover, an aircraft scatter was detected 202 times.

**Fig 26:**
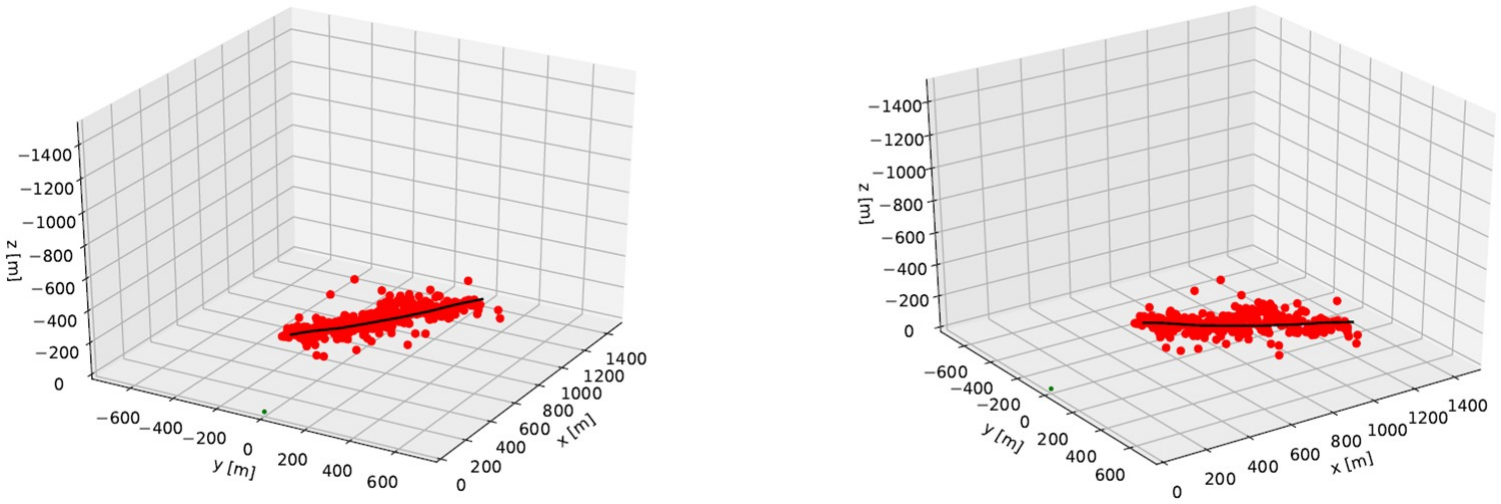
3D results for radar DoA estimation and GPS track, as seen from two different angles.

The DoA algorithm is applied to the detected pixels, and the result of that is plotted in Fig 26. The orange scatter points are the locations of the 3D detection, and the black line is the track of the aircraft, as logged by the GPS. The green dot is the location of the radar, which is plotted on the point (0, 0, 0). The x-axis points horizontally in the looking direction of the radar. Both subfigures in Fig 26 contain the same data, only plotted from a different angle.

**Fig 27:**
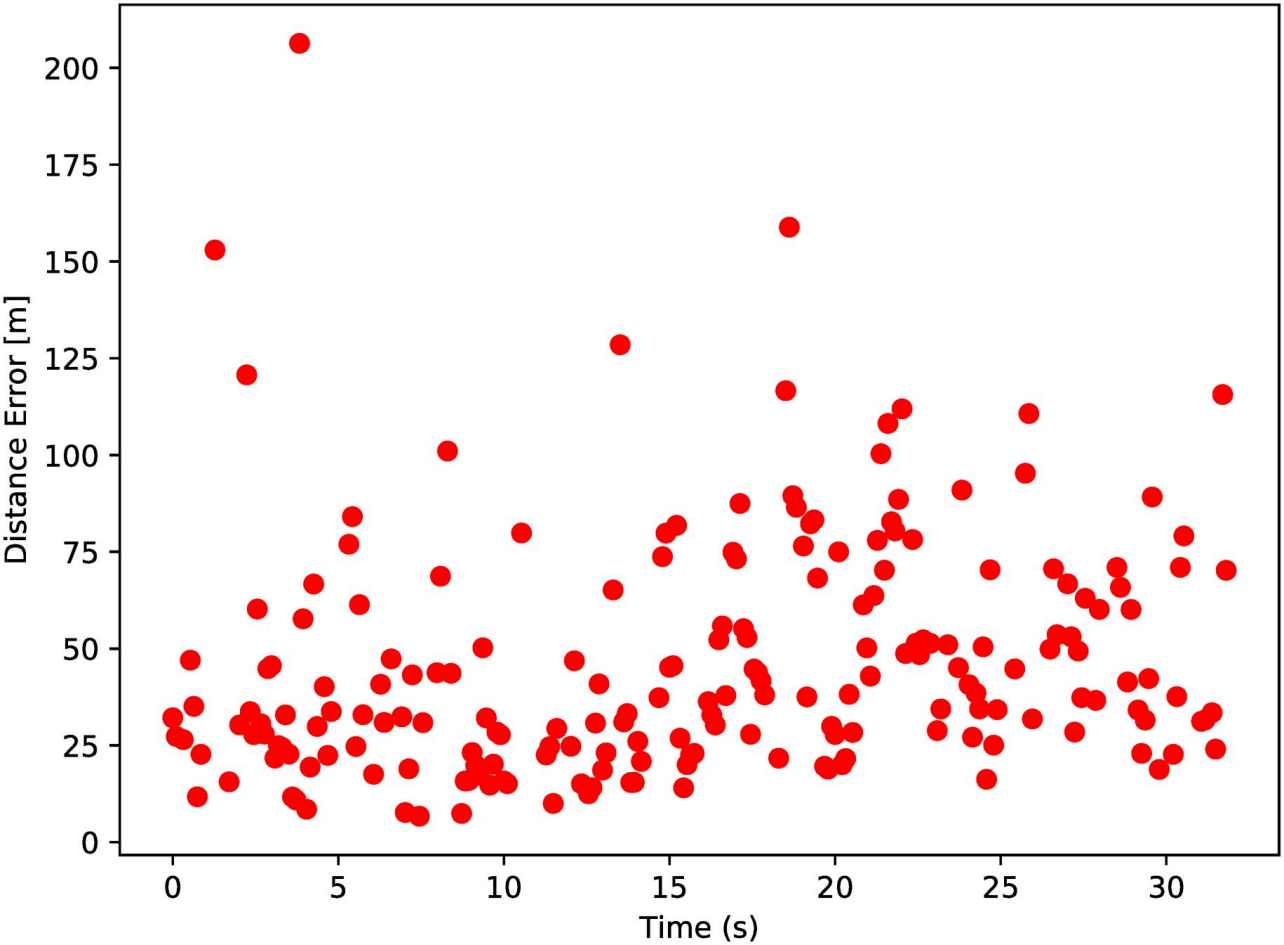
Distance from radar scatters to GPS position.

As can be seen in Fig 26, the results for the DoA form a cloud along the GPS track of the aircraft. The scatters appear to be accurate in following the aircraft, but this needs to be quantified. Therefore the distance from the DoA estimates to the aircraft position is plotted as well, of which the results are seen in Fig 27.

**Fig 28:**
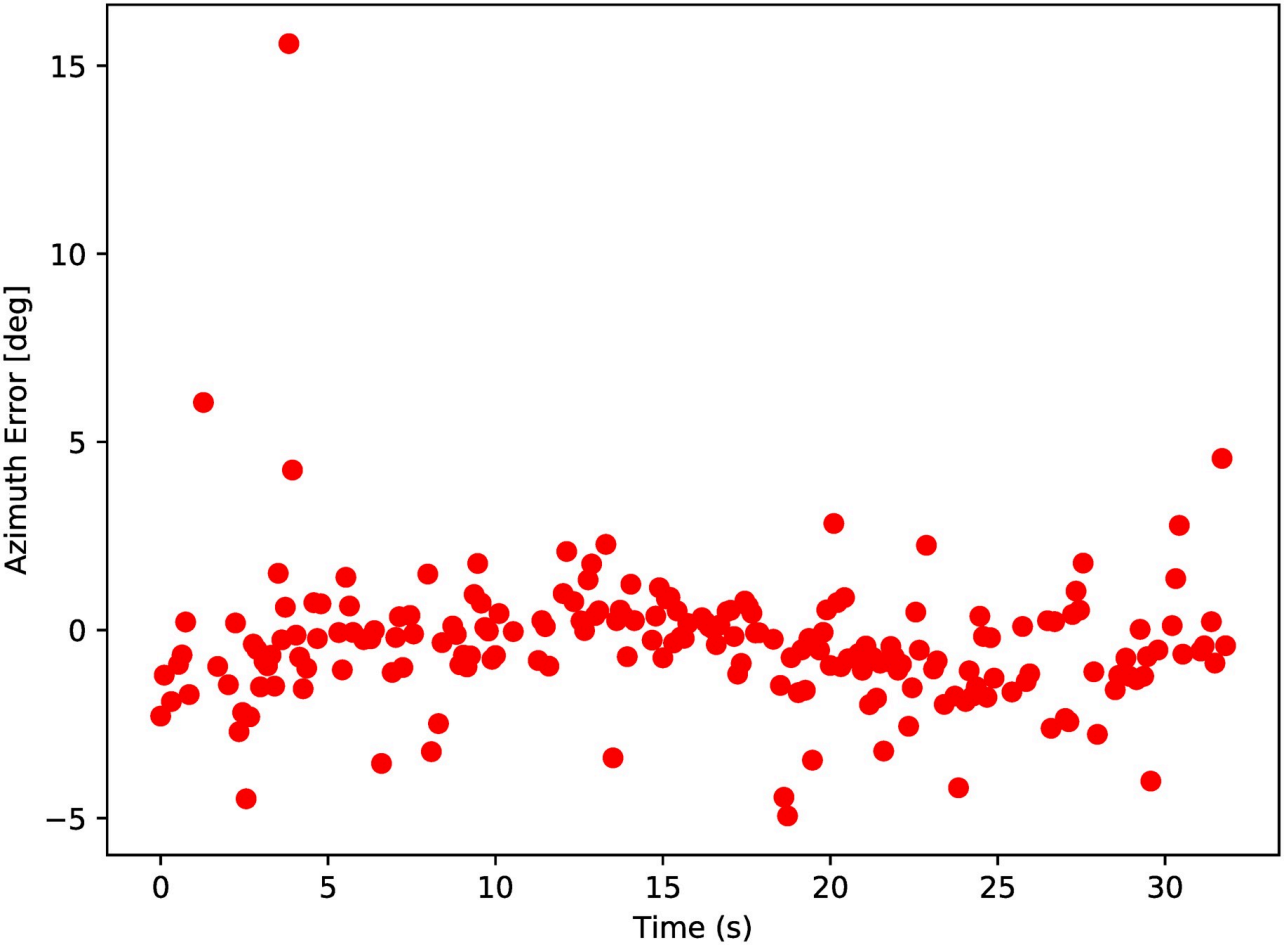
Azimuth and elevation differences between radar and GPS results.

From the data in Fig 26 it is seen that the points are above and below the actual aircraft tracks. Fig 27 however, does not provide an indication about the direction of the distance to aircraft position, only the absolute value. In order to indicate the value of this, the distance between the scatter and GPS locations are also expressed in azimuth and elevation errors, as seen from the radar point of view. The results are given in Fig 28.

**Fig 29:**
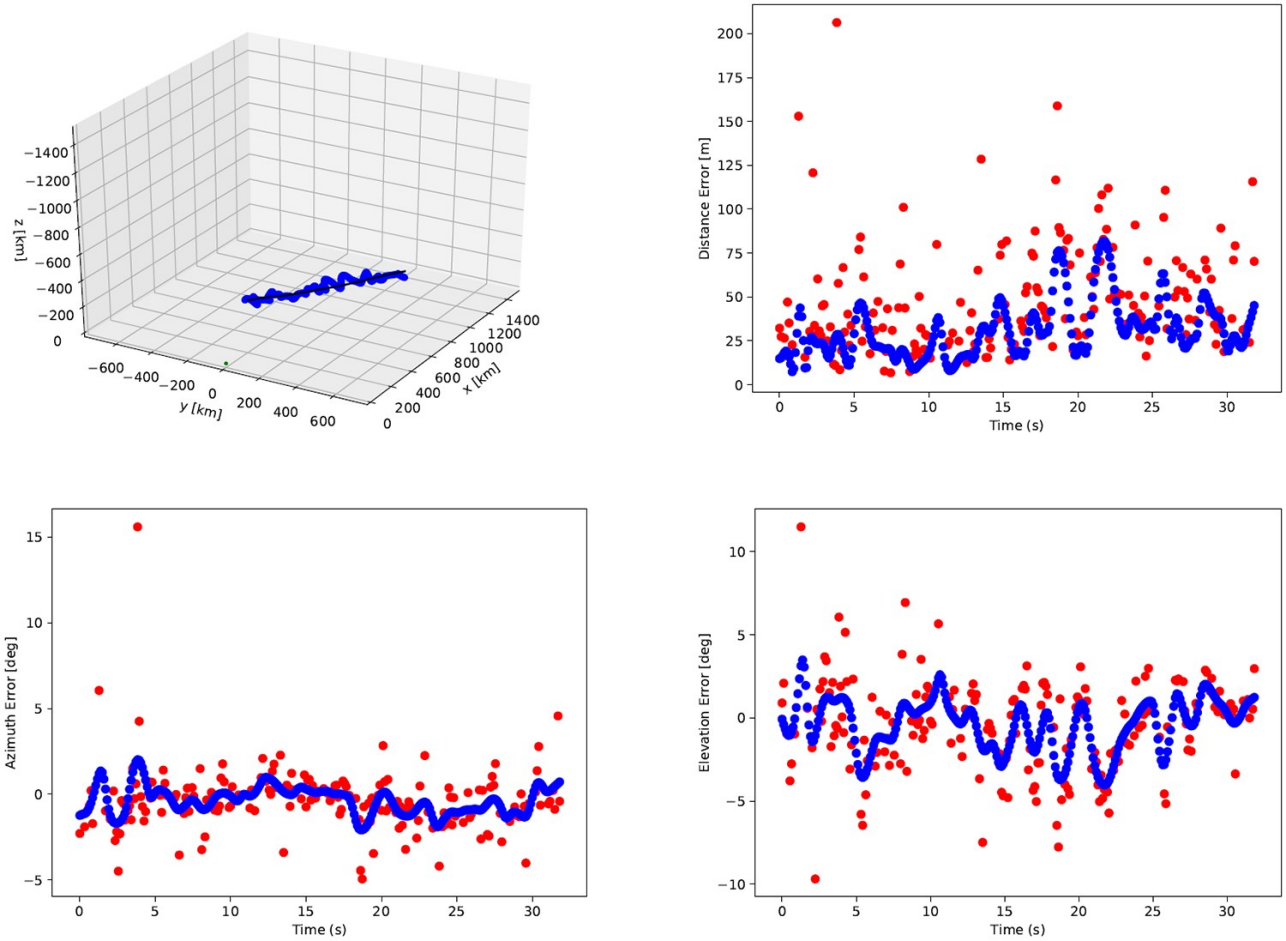
3D results before (red) and after (blue) application of Hanning window function.

As seen from Fig 28, high-frequency noise appears to cause errors on the measurements. This means that a low-pass filter may be used to remove the high-frequency components of the results. A simple Hanning filter [35] is therefore applied on the 3D DoA results in Fig 26, and the results of this are given in Fig 29.

In order to quantify the accuracy of the 3D algorithm, the mean and standard deviation are computed for the distance, azimuth and elevation errors, both for the raw data and the hamming filtered results. These values can be found in Table 2.

**Table 2:** Mean (*μ*) and standard deviation (*σ*) of difference between radar and GPS results.

	Raw		Hanning	
	*μ*	*σ*	*μ*	*σ*
distance [*m*]	46.2	30.3	30.7	16.1
azimuth [*deg*]	-0.4	1.8	-0.37	0.8
elevation [*deg*]	-0.5	2.7	-0.43	1.6

## 6 Discussion

In section 2.1, the appearance of objects in a radar image was discussed. It was found that objects are expected to take shape as blurred pixels in the radar image. When the first field test was performed, it was found that this indeed was the case, as was seen in Fig 24. It was confirmed that the radar was possible to track the reflection of the aircraft, after spurious signals were removed from the image.

### 6.1 Radial velocity results

When the tracked values for the range and radial velocity are compared to those of the GPS measurements, it is seen that the trends in both figures are similar (Fig 25). However, it is seen immediately that the blue line in the radial velocity plot is varying around the red pixels. Since the variations are so abrupt (the big spike is a difference of 10*m*/*s* in one second: a sudden deceleration of 1g), it is reasonable to assume that the red scatter points describe the radial velocity more accurately.

For the blue line, the GPS radial velocity, it should be stated that this velocity can only be computed indirectly: GPS can pinpoint the location of an object and its velocity can be computed by subtracting consecutive measurements. For the radar, the radial velocity is computed directly, by taking the Doppler shift. The difference in *V*_*R*_ accuracy can also be explained because the location measurement from the GPS typically is accurate within several meters, but one pixel in the radar image is 0.075*m*/*s* wide. It can therefore be said that the accuracy of the radar *V*_*R*_ measurements is excellent with respect to that of GPS.

### 6.2 Range results

For the range measurements, which are also presented in Fig 25, it is also seen that the radar yields results similar to GPS. The typical position errors of several meters of GPS have little effect on the results, since the scale of the range measurements is of hundreds of meters. Also the pixel size in the radar image (one pixel is 15*m* long) is of little influence. It is seen that the radar results follow the GPS line closely, but that later in the experiment the differences between radar and GPS become larger.

Several explanations can exist for this. It is possible that the radar has an offset, that the results are biased at larger distances. It is also possible that the timing between the GPS and radar clocks was off by about one second; if the blue line would move to the right with respect of the red line the differences would also become smaller. This would have an effect predominantly in the later part where the line gradients are higher.

A third option is that the position of the ground station was measured inaccurately (by GPS) and was off by a few meters. Any location that is closer to the end of the flyover but at the same distance from the start of the experiment would yield better results to the red line.

From this experiment, it cannot yet be concluded which of these explanations causes the differences in range measurements.

### 6.3 3D positioning

After the first field test, if was concluded that it is possible to calibrate the radar system and to perform 3D Direction of Arrival Estimation, with which the location of an object in 3 dimensions can be determined. Now that the calibration and DoA estimation are applied to the flyover, it is found that indeed the 3D radar scatters follow the flight path of the aircraft over the test location.

### 6.4 Accuracy

It is computed that the 3D scatter points are on average 46.2*m* removed from the true GPS location, with a standard deviation of 30.3*m*. The GPS results may have been a few meters off, as discussed in the section above. This would affect the *μ* of 46.2*m*, but would have a minor effect on the standard deviation.

The average distance between GPS and radar results is an absolute distance and can therefore never become negative. More information about the accuracy of the DoA algorithms can be found when the results are expressed in azimuth and elevation angles. It is found that the standard deviations for azimuth and elevation errors are 1.8 and 2.7 degrees, respectively. The Hanning filter reduced those values to 0.8 and 1.6 degrees. This can be sufficient to provide a mobile ground station with an image of objects in the sky above. The mean errors have values of 0.4 and 0.5 degrees, which may be caused by tiny misalignments of the radar: the equipment to measure the radar attitude was accurate to a single degree, so smaller errors such as these can be fixed with accurate calibration of the radar platform.

The accuracy of the DoA algorithm can be improved by raising the number of receiving antennas. In this experiment, only 4 receiving antennas were used, but it is possible to increase that number if a better accuracy is required. The number of equations for the linear least squares problem in section 3.2 increases if more antennas are added to the setup. Having more antennas can also decrease the effect when a single antenna measurement is disturbed. It is remarkable that the DoA algorithm yields more accurate results in azimuth direction than in elevation direction. The antennas formed a square 2x2 pattern, which is symmetrical in horizontal and vertical direction. The only difference can be the polarization direction of the radar signals. It is unclear whether the difference in azimuth and elevation accuracy originates from the radar system, or from the test environment.

### 6.5 Range limit

The range limit of the radar system is unknown, since it is not tested explicitly. The largest distance that the aircraft had to the ground station was just over 3*km*, at which the radar reflection was visible in the radar images, but only when they are viewed in a sequence—the signal was too weak to be differentiated from background noise when just a single image was observed. Novel visual tracking algorithms are able to detect such reflections when the data is treated as a streamed video, so it can be possible to detect even these reflections. A larger number of antennas in the configuration can also help to increase the sensitivity, and increase range for the radar. The maximum attainable range is furthermore dependent on the radar cross-section of the object. Larger aircraft will be visible from larger distances.

### 6.6 Comparison to other products

It is important to compare the performance of the FMCW radar to that of Flarm devices and airport surveillance radars. The FMCW radar is portable and can be powered by a small battery, and can therefore be deployed at any location. The radar has a field of view of 80 degrees, in vertical and horizontal directions. In order to cover the entire sky, a constellation of multiple systems is required. An alternative is to have the radar pivot around an axis, similar to a primary radar. When objects are observed around the aircraft, the radar can pinpoint the direction of the signal source within a few degrees accuracy. This is significantly better than e.g. Flarm, which can localize an object in 12 segments of 30 degrees in horizontal direction, and 5 vertical layers relative to the device. Multiple consecutive measurements can further improve the accuracy, as the high-frequency noise can be countered with a low-pass filter. The radar system can detect various objects, independent on whether they carry the proper equipment. The radar results are dependent on the radar cross-section of the objects. A Pipistrel Virus 912 aircraft was visible up to 3*km* distance.

The experiment confirms that the radar can be used to detect aircraft within the vicinity of the radar. Future research is needed to test whether the radar can be used while in motion, so whether it may be used in the air. It is also required to test the radar in rotating mode, in order to observe the complete environment. Additional techniques to filter reflections based on their elevation can help to separate aircraft from ground reflections.

## 7 Conclusion & recommendations

In this paper, research was presented towards the possibility of building a portable primary radar for General Aviation. This is empowered by the recent rapid development of radar hardware. Such a system has to be affordable, small and have a low power consumption, and the hardware tested in this paper matched those requirements. A test was performed with an aircraft flying over the radar, which could be observed in the radar image for a range up to 3*km*. The aircraft was tracked with an on-board GPS for a flyover at closer distance and the radar was able to detect the aircraft autonomously and to determine its location with an accuracy of on average 46*m*. The direction of the incoming signals can be determined within 2 degrees horizontally, and 3 degrees vertically. If the aircraft is tracked, low-pass filters can be applied to filter out the high-frequency noise and increase the accuracy of the three dimensional position estimates. Expanding the number of antennas beyond the 4 used in this research can also improve the radar results further. It is concluded that the development of portable primary radars may be an efficient means of increasing the situation awareness around the radar. Future research is required to develop both the hardware and software for aviation purposes, such as in-air use and separation of ground reflections.
